# Cultivar‐specific preference of bacterial communities and host immune receptor kinase modulate the outcomes of rice–microbiota interactions

**DOI:** 10.1002/imt2.70098

**Published:** 2025-12-16

**Authors:** Jiwei Xu, Peiyao Hu, Meng Liu, Wanyuan Zhang, Kabin Xie

**Affiliations:** ^1^ National Key Laboratory of Crop Genetic Improvement, Hubei Hongshan Laboratory, Huazhong Agricultural University Wuhan China; ^2^ Hubei Key Laboratory of Plant Pathology, Huazhong Agricultural University Wuhan China

**Keywords:** beneficial, cultivar preference, gnotobiotic cultivation, host immunity, microbiome, rice

## Abstract

Deciphering how plant–microbiota interactions achieve beneficial outcomes for crops will provide innovative strategies for sustainable agriculture. Here, we dissected rice‐microbiota dynamics using a tailored gnotobiotic cultivation system that models the semiaquatic environment in a paddy field. Inoculation with native soil microbiota resulted in root‐growth‐promotion (RGP) and root‐growth‐inhibition (RGI) phenomena in different cultivars. This preference persisted in a simplified synthetic community and individual bacterial strains, indicating that cultivar‐specific growth promotion is an intrinsic property of microbial inocula. Though stochastic process dominated the assembly of root microbiome in gnotobiotic cultivation, absolute quantification revealed that imbalance of detrimental and beneficial bacterial loads in roots correlated with RGP or RGI outcomes in different rice cultivars. From the host perspective, genetic screening identified that receptor‐like kinase mutants, including *OsFLS2* (*FLAGELLIN‐SENSITIVE 2*), inverted microbiota functionality, converting RGP to RGI. In particular, over 4534 rice genes responded to microbiota inoculation and 46.1% of them were reprogrammed in *osfls2* mutants, demonstrating the prominent regulatory role of *OsFLS2* in rice‐microbiota signaling. On the basis of these results, we propose that the rice‐microbiota relationships are gated by cultivar‐specific preferences of the bacterial microbiota and host immune receptor kinase, which provides a useful framework for crop microbiome engineering in the future.

## Highlights

- The same bacterial inocula display beneficial, neutral, or detrimental effects on the root growth of different rice cultivars.
- The imbalance of detrimental and beneficial bacterial loads in roots correlated with root‐growth promotion or inhibition outcomes in two rice cultivars.
- The conserved receptor‐like kinase *OsFLS2* is required to prevent microbiome dysbiosis.

## Graphical abstract

This study employed a tailored gnotobiotic cultivation system to investigate rice–microbiota interactions from both microbial and host perspectives. We found that the same bacterial inocula, including native soil microbiota, synthetic communities, and individual strains, exerted beneficial, neutral, or detrimental effects on rice root growth across cultivars. Our analysis suggests that a two‐tiered regulatory framework, comprising cultivar‐specific preference of the bacterial microbiota and host immune perception, governs the outcomes of rice–microbiota interactions.

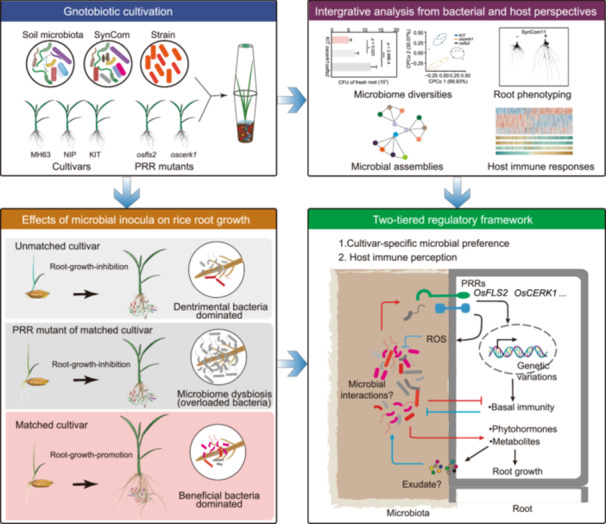

## INTRODUCTION

Plants host a multitude of microorganisms, referred to as the microbiota, in the rhizosphere, phyllosphere, and endosphere [1, 2], which modulate plant growth [3], disease resistance [4], nutrient absorption [5], and abiotic stress tolerance [6]. As primary interfaces for plant‐microbe interactions, roots recruit soil‐derived microbes via a two‐step model, wherein root exudates drive a shift in community composition in the rhizosphere, after which the plant host fine‐tunes the root endosphere communities [7, 8]. However, the complex interactions between crops and the rhizosphere microbiota remain unclear, hindering the application of beneficial microbiota in agriculture [9, 10, 11].

Recent advances in sequencing‐based metagenomics have demonstrated that the plant rhizosphere and phyllosphere microbiomes are shaped by host genetic variations. Integrating metagenomics and genome‐wide association studies (GWAS) uncovered correlations between microbiome diversity and host genetics across multiple species, including *Arabidopsis thaliana* [12], rice (*Oryza sativa*) [13, 14], foxtail millet (*Setaria italica*) [15], maize (*Zea mays*) [16], switchgrass (*Panicum virgatum*) [17], and barley (*Hordeum vulgare*) [18]. Several studies have further identified the specific host genes driving microbiome variations. For instance, two rice subgroups, *indica/xian* and *japonica/geng*, assemble distinct rhizosphere microbiomes, and the recruitment of a large proportion of *indica*‐enriched bacteria is associated with a nitrate transporter/sensor *NRT1.1B* [13, 19]. Recent microbe GWAS revealed that rice *PHENYLALANINE/TYROSINE AMMONIA LYASE* (*OsPAL02*) regulated rice phyllosphere microbiome homeostasis by controlling the synthesis of 4‐hydroxycinnamic acid [14]. A microbe GWAS using 129 maize accessions revealed that the abundance of *Massilia*, a beneficial bacterium that promotes maize growth under low‐nitrogen conditions, was associated with variations in a single host gene [16]. These findings collectively underscore how host genetic determinants critically govern rhizosphere microbial assembly, providing potential strategies for precision microbiome engineering [20] to enhance agricultural sustainability.

Plant innate immunity, notably microbe‐associated molecular pattern (MAMP)‐triggered immunity (MTI), surveils all microbes (pathogenic, beneficial, and commensal), even though its primary role is considered pathogen defense. Plants employ receptor‐like kinases (RLKs) as pattern recognition receptors (PRRs) to recognize bacterial MAMPs and activate MTI responses [8, 21]. In addition to defending against microbial pathogens, MTI is involved in the prevention of microbiome dysbiosis, which dampens plant health [22, 23]. Excess immune response costs plant growth, whereas beneficial bacteria have evolved various strategies to intercept the MTI response. For example, commensal bacteria modulate auxin homeostasis and defense‐related gene expression in *Arabidopsis*, resulting in the overriding of bacterial flagellin (i.e., the flg22 epitope)‐triggered root growth inhibition (RGI) [3, 24, 25, 26]. However, our knowledge of the interactions between plant immunity and microbiota is largely from the model species *Arabidopsis thaliana*. Crops, such as rice, encode approximately twice as many RLKs as the *Arabidopsis* genome does, but only a few RLK PRRs involved in bacterial and fungal pathogen recognition have been characterized.

This raises the question of whether and to what extent host plants, including their genetic variations and the conserved immune systems, affect the effects of the soil microbiota and bioinoculants. To address this question, we developed a gnotobiotic cultivation system that models the semiaquatic environment of a paddy field. From the microbial perspective, we observed that a native soil microbiota (referred to as NSM hereafter) has robust root growth‐promoting effects on the elite *indica* rice cultivar Minghui63 (MH63), which has been widely used in rice breeding in China [27]. Interestingly, the same soil microbiota had beneficial, detrimental, or neutral effects on the growth of the roots of 10 different rice cultivars, revealing the cultivar‐preference of microbial inocula. From the host plant perspective, we detected global transcriptomic response in rice upon NSM inoculation. Further analysis indicated that *OsFLS2* is required for microbiota‐triggered root‐growth‐promotion (RGP) and for the transcriptional suppression of defense‐related genes. These findings indicate that the outcomes of rice–microbiota interactions are gated by cultivar preferences of the microbiota and host immunity. Our data provides a conceptual model of rice‐microbiota interactions integrating both microbial and host perspectives, which will help in the engineering of beneficial microbiomes for crops.

## RESULTS

### Native soil microbiota promotes the root growth of the rice cv. MH63

**Figure 1:**
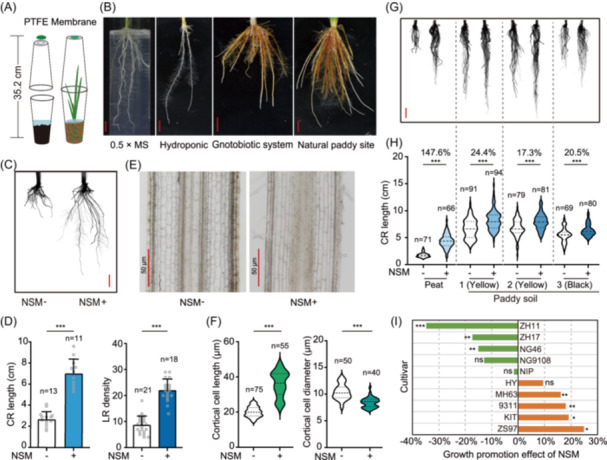
Effects of native soil microbiota (NSM) on rice root growth under gnotobiotic conditions. (A) Schematic illustration of gnotobiotic bottles for rice cultivation. The gnotobiotic bottle contained sterilized soil with flooding water to model the paddy field conditions. The membrane in the lid allows the exchange of air. See also Supplementary Note 1 and Figure S1. (B) Photos of rice roots growing in 0.5 × MS agar media, hydroponic culture media, gnotobiotic bottles, and natural paddy fields. The photos were taken at 14 days after germination. (C, D) Comparisons of the crown root (CR) length and lateral root density (LR) of MH63 rice in axenic (NSM−) and NSM‐inoculated soils (NSM+). (E) Longitudinal sections showing the cell size of rice roots. (F) Violin plots showing the length and diameter of cortical cells of rice roots in axenic (NSM−) and NSM‐inoculated soils (NSM+). (G, H) Evaluation of NSM effects in different soils. Photos were taken at 14 days post inoculation (G). NSM‐stimulated root growth relative to that of axenic plants is shown as a percentage at the top (H). Paddy soil 1/2 (yellow) and 3 (black) were collected in central and northern China, respectively. (I) Effects of NSM on the growth of the roots of 10 rice cultivars. The growth‐promoting effects are shown as the relative increases/decreases in root length after inoculation with NSM. The full names and abbreviations of the rice cultivars are as follows: *indica* cultivars: MH63, Minghui63; ZS97, Zhenshan97; 9311, 93‐11; *japonica* cultivars: KIT, Kitaake; HY, Hwayoung; NIP, Nipponbare; NG9108, Nangeng9108; NG46, Nangeng46; ZH17, Zhonghua17; and ZH11, Zhonghua11. For B, C, G, Bars = 1 cm; for E, Bar = 50 μm. For F, H, statistical analyses are performed using the Kruskal–Wallis test. For D, data are presented as mean ± standard deviation (SD). Student's *t*‐test, *n*, sample size. *p < 0.05; **p < 0.01; ***p < 0.001; ns, no significant difference.

To investigate rice microbial interactions in semiaquatic paddy fields, we designed a gnotobiotic bottle to simulate the anaerobic growth conditions of natural fields (Figures 1A and S1A−D, Supplementary Note 1). This gnotobiotic system can support rice growth for up to 10 weeks and allow short‐life‐cycle rice cultivar flowering and seeding inside (Figure S1E−J). We compared this gnotobiotic system with agar media and hydroponic solution cultivation systems using *indica* cultivar MH63. After inoculating NSM from a paddy field, the rice plants in gnotobiotic bottles developed root architecture and reddish‐brown iron plaques similar to those in a natural paddy field (Figure 1B), which are critical for root‐bacteria interactions in aquatic plants [28]. In contrast, rice roots in agar media or hydroponic solution have distinct architectures and lack iron plaque (Figure 1B), implying that these two rice culture systems differ from the paddy field conditions. Furthermore, the number of culturable bacteria in the soil of the gnotobiotic bottles reached 4.39 × 10^6^ colony‐forming unit (CFU)/g soil at 14 days after the inoculation of NSM, which is comparable to that of the paddy field (5.86 × 10^6^ CFU/g soil; Figure S1F), further demonstrating that this gnotobiotic bottle establishes a microbial ecological condition similar to a paddy field. Thus, this gnotobiotic bottle was used hereafter to study the interactions between rice and the soil microbiota.

To assess the effects of the soil microbiota on rice root traits, NSM was applied to germ‐free germinated MH63 seeds growing in autoclaved organic soils in gnotobiotic bottles. After 14 days, the root systems of the NSM‐inoculated rice plants were markedly distinct from those of the axenic plants (Figure 1C). Compared with those under the axenic treatment, the crown root length and lateral root density of the rice plants in the NSM‐inoculated soil increased 2.69‐ and 2.52‐fold, respectively (Figure 1C,D; Student's *t* test, *p* < 0.001). Histological analysis revealed that the lengths of epidermal and cortical cells in the elongation and differentiation zones of roots increased in NSM‐inoculated soil (Figure 1E,F). This RGP effect on MH63 rice was consistently observed when different batches of NSM preparations were used, implying that RGP is a common trait of the soil microbiota for MH63 rice.

### Soil type and rice variety affect the RGP traits of the soil microbiota

Given the profound effects of soil phytochemical properties on root architecture [29] and microbiome assembly, we evaluated the RGP activity of NSM on MH63 rice using peat soil and three paddy soils collected from central China (soil #1 and #2, yellow) and northern China (soil #3, black). These paddy soils were obtained from different rice production regions and exhibited differences in texture, compactness, organic matter, and available inorganic nutrients (Figure S2A,B). Rice roots grown in yellow paddy soil were 21.3% to 279.2% longer than those in black and peat soils, respectively, regardless of whether they were germ‐free or subjected to NSM inoculation (Figure 1G,H; two‐way ANOVA followed by post hoc Tukey's test, *p* < 0.0001), which is consistent with previous reports that soil texture and compactness affect root elongation [29]. Soil type and NSM inoculation explained 54.6% and 10.06% of the total variance in root length, respectively (soil: *F*_(3,520)_ = 285; NSM: *F*_(1,520)_ = 157.5; all *p* < 0.001). Compared with those in the germ‐free treatment, the rice root length increased by 17.3% (paddy soil #2) to 147.6% (peat soil) in the NSM‐inoculated soils (Figure 1G,H), suggesting that NSM affected the RGP to different extents in these four soils.

To investigate the effects of the host genotype on the RGP trait of NSM, we examined the root length of 7 *japonica* and 3 *indica* cultivars after NSM inoculation under gnotobiotic conditions using paddy field soil #2 (Figure S2C−E). The rice genotypes and their interaction with NSM explained 34.99% and 12.29%, respectively, of the total variance in root length (Figure 1I; two‐way ANOVA followed by post hoc Tukey's HSD test, genotype: *F*_(9,316)_ = 22.96, NSM: *F*_(9,316)_ = 8.07, all *p* < 0.001). Compared with those under axenic growth conditions, the microbiota stimulated root elongation by 16.1%−24.2% in 3 *indica* cultivars (MH63, 9311, and ZS97) and 1 *japonica* cultivar (KIT) (Figure 1I; Student's *t*‐test, *p* < 0.05). In contrast, NSM inhibited root growth by 15.1%−34.8% in 3 *japonica* cultivars (ZH11, ZH17, and NG46). The lengths of the roots of the *japonica* cv. Nipponbare (NIP), HY, and NG9108 plants did not significantly differ between the axenic and NSM‐inoculated soils. Overall, we found that the same microbiota inoculant had beneficial, detrimental, and neutral effects on the root growth of different cultivars (Figure 1I), indicating the deterministic role of rice genotype in the function of microbial inoculants.

### MH63 and KIT plants differentially assembled beneficial microbiota from NSM

**Figure 2:**
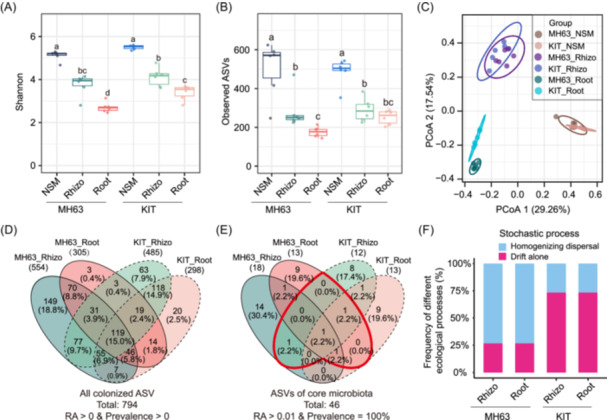
Assembly of bacteria from NSM in gnotobiotic cultivation. The germ‐free MH63 rice plants were grown in gnotobiotic bottles and inoculated with freshly prepared NSM (Input). Rhizosphere and root samples (six biological replicates) were collected at 14 days post‐NSM inoculation and analyzed via 16S rRNA gene amplicon sequencing. (A, B) Comparison of the α‐diversities of the input, rhizosphere (Rhizo), and root microbiota. The Shannon index (A) and observed ASV number (B) are presented. Different letters above the boxes indicate significant differences between experimental variants, as determined by the Kruskal–Wallis test followed by the Mann–Whitney *U* test. (C) Principal co‐ordinates analysis (PCoA) in combination with PERMANOVA on Bray–Curtis dissimilarities showing that the input, rhizosphere and root microbiota separated from each other (PCoA1) and were secondarily separated by cultivar (PCoA2). The variances explained by each axis are shown in brackets. Venn diagrams showing all colonized ASV (D) and ASVs of core microbiota (E) of rhizosphere and root between rice MH63 and KIT. The number in brackets indicates ASV number. The ASVs shared by core microbiota of two cultivars are highlighted with red line. (F) The frequency of different ecological processes of each community assembly cue was defined as the proportion governed by each process. According to Jizhong Zhou and Daliang Ning [30], dispersal is defined as “movement and successful colonization (establishment) of an individual organism from one location to another via both active and passive mechanisms”; drift is defined as “random changes, with respect to species identity, in the relative abundances of different species within a community over time due to the inherent stochastic processes of birth, death, and reproduction.”

In this gnotobiotic culturation experiment, the rhizosphere and root microbiota were recruited from the input inocula, providing a tractable system to investigate microbial community assembly. To profile bacterial compositions, we performed 16S rRNA gene amplicon sequencing on the initial NSM inocula (Input) and the rhizosphere and root communities of two rice cultivars (MH63 and KIT, 6 biological replicates per cultivar) which inoculated with input communities for 2 weeks. After filtering low‐abundance amplicon sequence variants (ASVs), a total of 1104 ASVs were obtained from 36 samples (Table S1). Consistent with field observations of reduced bacterial diversities from soil to roots, the α‐diversity metrics, including ASV richness and Shannon indices, progressively declined from the input inocula to rhizosphere and root communities (Figure 2A,B). Principal coordinates analysis (PCoA) shows that rhizosphere and root communities diverged significantly from the input inocula, with further separation between root communities of two cultivars (Figure 2C; permutational multivariate analysis of variance (PERMANOVA), cultivar: *R* ^2^ = 0.15, *p* < 0.001; compartment: *R* ^2^ = 0.46, *p* < 0.001), implying that host genotype influenced the root microbiota structure. At the phylum level, rhizosphere communities in both cultivars were dominated by Pseudomonadota (36.8%–64.6%) and Bacteroidota (20.6%–30.1%), whereas the root communities were overwhelmingly composed of Pseudomonadota (95.7%–98.4%). Among the 794 ASVs detected in root and rhizosphere samples, 46.7% of them colonized both cultivars (Figure 2D). Based on the ASV rank‐abundance relationships (Figure S3) and previous studies [31], we defined core microbiota as ASVs with relative abundance (RA) > 0.01 (1%) and 100% prevalence across six replicates. This stringent definition identified core microbiota comprising only 12–18 ASVs, with four ASVs shared between the core microbiota of MH63 and KIT (Figure 2E). Despite this limited taxonomic diversity, the core microbiota account for a substantial portion (53.7%–76.5%) of the total microbial abundance in both rhizosphere and root communities (Figure S3). These results imply that under gnotobiotic conditions, the core microbiota of rice plants is dominated by a small, taxonomically distinct set of strains, with limited overlap between cultivars.

The assembly of microbial communities is shaped by a continuum of deterministic and stochastic processes, including selection, dispersal, diversification, and drift [30, 32]. We applied the phylogenetic bin‐based null model (iCAMP) [33] to quantitatively assess the ecological processes governing bacterial community assembly. For both cultivars, the β‐nearest taxon indexes of root and rhizosphere communities ranged between −2 and 0, indicating that stochastic processes predominantly governed community assembly (Figure 2F and Figure S4). These stochastic processes are further divided into probabilistic dispersal and undominated processes based on the Bray–Curtis‐based Raup–Crick index (RC_bray_). Notably, the RC_bray_ values differed significantly between two cultivars (Figure S4). In MH63, microbial diversities were primarily driven by homogenizing dispersal (RC_bray_ < −0.95); whereas in KIT, the ecological drift (−0.95 < RC_bray_ < 0.95) emerged as the dominant driver (Figure 2F and Figure S4). Consistently, the MH63 microbial communities exhibited tighter clustering than those of KIT, a pattern consistent with the homogenizing dispersal process [30]. These findings suggest that, despite both cultivars exhibiting RGP after inoculating NSM, the assembly of their root microbiomes in gnotobiotic cultivation was dominated by different ecological processes.

### A SynCom consisting of 11 strains recapitulates the RGP traits with altered cultivar preference

**Figure 3:**
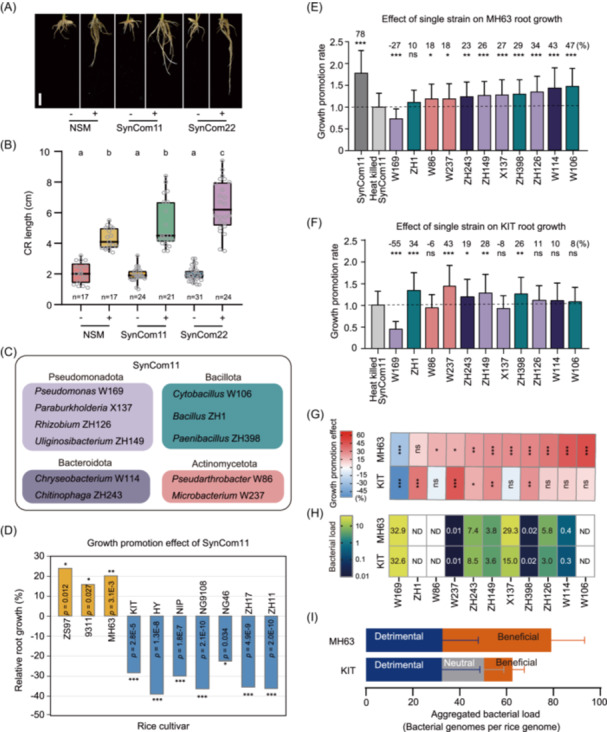
A SynCom with 11 strains recapitulates cultivar‐specific RGP. (A, B) RGP effects of NSM, SynCom22, and SynCom11 on MH63 rice root growth. Different letters denote significant differences (one‐way ANOVA followed by Duncan's multiple range test, *p* < 0.05); *n*, sample size; −, filter‐sterilized NSM or heat‐killed SynCom; +, live NSM/SynCom. Bar = 1 cm. (C) Bacterial composition of SynCom11. (D) Effects of SynCom11 on the root growth of 10 cultivars. The growth‐promoting effects are presented as the relative increases/decreases in root length after inoculation with NSM. (E, F) Comparisons of the RGP effects of individual strains of SynCom11. MH63 and KIT rice plants were inoculated with individual bacterial strains and SynCom11, and heat‐killed SynCom11 was used as a mock control. The RGP and RGI effects (%) are shown as changes in relative root growth in comparison with the control (*n* = 10−24). (G) Root growth promotion effects of a single strain on MH63 and KIT, respectively. The effects are defined as the relative increases/decreases in root length after inoculation with corresponding bacterial strains. (H) Bacterial load of each strain of MH63 and KIT after being inoculated with SynCom11. ND, not detected. See Figure S8 for qPCR data and statistical analysis. (I) Aggregated bacterial load of detrimental, neutral and beneficial strains in MH63 and KIT roots. ns, no significant difference; *p < 0.05; **p < 0.01; ***p < 0.001 (Student's *t*‐test).

Given high compositional complexity and low trackability of NSM, we investigated whether microbiota‐mediated RGP could be replicated using a simplified community. To this end, we employed a bottom‐up strategy [34, 35], beginning with 6795 bacterial isolates obtained from rice roots in paddy fields via high‐throughput bacterial cultivation [36]. These isolates represented 40.4% (61/151) of the prevalent bacterial families in native rice root communities (Figure S5A). From 677 isolates with high purity (>99%) in high‐throughput bacterial cultivation [36], we randomly selected two strains per family and retained the fastest‐growing isolate per pair, resulting in a 22‐strain SynCom (SynCom22) spanning 13 bacterial families (Figure S5B). Remarkably, SynCom22 displayed a stronger RGP effect on cultivar MH63 than NSM did (Figure 3A,B). To further simplify this community, we randomly excluded 50% of strains, resulting in an 11‐strain SynCom (SynCom11) representing four dominant phyla in rice root microbiota (Figure 3C, Table S2, and Supplementary Note 2 for SynCom design). SynCom11 displayed RGP effects on MH63 comparable to NSM (Figure 3A,B), supporting our hypothesis that a small number of taxonomically diverse bacteria suffices to replicate beneficial microbiota functionality.

Interestingly, SynCom11 displayed cultivar‐specific RGP that diverged from NSM when tested across 10 rice cultivars. Compared to axenic controls, SynCom11 promoted root growth in 3 *indica* cultivars (MH63, ZS97, and 9311) but suppressed growth in 7 *japonica* cultivars (KIT, HY, NIP, NG46, NG9108, ZH11, and ZH17) (Figure 3D and Figure S6), suggesting that cultivar preference is an inherent property of microbial communities. Strikingly, SynCom11 and NSM exhibited distinct effects on 2 cultivars: SynCom11 impairs root growth in KIT and NIP, whereas NSM enhanced in KIT and had no effect on NIP (Figures 1I and 3D). This discrepancy suggests that SynCom11 does not fully replicate the functional complexity of NSM, implying that cultivar‐specific RGP was probably determined by taxonomic composition and functional interactions within the consortium.

### The opposite effects of SynCom11 are associated with unbalanced loads of beneficial and detrimental bacteria

The change of cultivar preference of SynCom11 allowed us to investigate the assembly mechanisms underlying beneficial and detrimental microbiota formation. To trace functional contributors, we assessed the effects of individual SynCom11 strains on rice root growth of cultivars MH63 and KIT (Figure 3E–G). Single‐strain inoculation assays revealed that 9 strains (W86, W237, ZH243, ZH149, X137, ZH398, ZH126, W114, and W106) promoted root growth (beneficial), 1 strain (W169) suppressed growth, and 1 strain (ZH1) showed no effect on MH63 (Figure 3E). In cultivar KIT, 5 MH63‐beneficial strains (W86, X137, ZH126, W114, W106) became neutral, while strain ZH1 shifted from neutral to beneficial. These findings confirm that SynCom11 harbors cultivar‐specific functional strains driving RGP/RGI outcomes for two cultivars.

To resolve strain interactions in SynCom11, we performed binary inhibition assays [22], where a target strain plated on agar was challenged by an attacker strain. Among 110 strain combinations, 24 inhibitory interactions were identified (Figure S7). Notably, 70.8% (17/24) of inhibitory interactions involved ZH1 or W169 as attacker strains. Though the high‐order interactions were not resolved in this experiment, our data imply the antagonistic binary interactions between beneficial strains and detrimental/neutral strains.

We further quantified SynCom11 colonization in rice roots. After inoculating SynCom11, 16S rRNA gene amplicon sequencing identified eight strains stably colonized rice roots. Among these bacteria, only three strains (W237, ZH398, and ZH149) exhibited significantly different relative abundances (strain reads/total reads) between MH63 and KIT (Figure S8; student's *t* test, *p* < 0.05). However, all three strains displayed identical RGP effects on both cultivars and occupied low relative abundances (0.01%−6%), indicating that shifts in relative abundance alone cannot explain the opposite effect of SynCom11 on two cultivars.

Given the critical role of microbial load in microbiome functionality [37, 38], we performed qPCR‐based absolute quantification using strain‐specific primers and quantified the load of each strain as bacterial genome copies per rice genome (Figure 3H and Figure S8). This approach detected eight root‐colonizing strains, with bacterial load ranging from 0.01–32.9 bacterial genome copies/rice genome. Notably, the detrimental strain W169 dominated root microbiomes and showed no differences between MH63 and KIT roots. Intriguingly, strains X137 and ZH126, which displayed RGP on MH63 but had a neutral effect on KIT, colonized MH63 roots at twice the load observed in KIT. We further categorized the colonized strains to beneficial, neutral, and detrimental based on single‐strain inoculation assays (Figure 3E–G), and calculated the aggregated bacterial loads of each catalog. In MH63, the aggregated load of beneficial bacteria (average = 46.75) exceeded detrimental bacterial loads (average = 32.89) by 1.42‐fold. Conversely, in KIT, detrimental bacterial loads surpassed beneficial bacteria by 2.7‐fold (Figure 3I). This imbalance aligns with SynCom11's contrasting effects: RGP in MH63 (beneficial‐dominated) versus RGI in KIT (detrimental‐dominated). These results imply that SynCom11's functional outcomes are dictated by the balance of beneficial and detrimental bacterial loads within the root microbiome.

### Rice PRR mutants subverted the RGP effects of NSM

**Figure 4:**
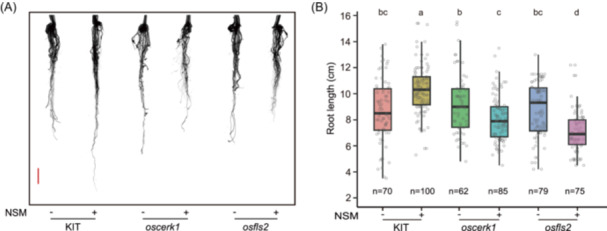
*OsFLS2* and *OsCERK1* are required for NSM‐triggered RGP. KIT, *oscerk1* and *osfls2* rice plants were inoculated with (+) or without (−) NSM for 14 days in gnotobiotic bottles and their root phenotypes were compared. (A) Photos of rice roots inoculated with and without NSM in gnotobiotic bottles. Bar = 1 cm. (B) Comparisons of the lengths of the roots of the KIT and mutant plants after inoculation with NSM. The results from three independent inoculation experiments are shown. Different letters denote significant differences (one‐way ANOVA followed by Duncan's multiple range test, *p* < 0.05). *n*, sample size.

Beyond characterizing microbial composition, we sought to understand how a beneficial microbiota promotes root growth in the presence of host immunity. We focused on rice group XII LRR‐RLKs and LysM‐RLKs due to their central roles as extracellular receptor/coreceptor of MAMP and their enrichment in microbiota‐responsive genes [39]. After screening a KIT‐background RLK mutant library [40] (Figure S9), we identified 4 mutants that subverted the RGP effects of NSM, namely, *osfls2* (LOC_Os04g52780), *chitin elicitor receptor kinase 1* (*oscerk1*, LOC_Os08g42580), and two LRR‐RLKs (LOC_Os02g12400 and LOC_Os12g42520) homologous to *elongation factor Tu receptor* (*EFR*). Among these 4 genes, we prioritized *OsFLS2* and *OsCERK1* for further analysis because *OsFLS2* is a classical PRR that recognizes flagellin [41, 42] and *OsCERK1* is one of the central regulators of plant immunity [43, 44]. In germ‐free soil, the root length of *osfls2* and *oscerk1* mutants was comparable to those of the wild‐type (WT, cv. KIT) plants. While NSM enhanced WT root length by 18.5%, which is within the ranges as we previously observed (Figure 1I), it inhibited root growth in *osfls2* and *oscerk1* mutants by 11.8% and 18.9%, respectively (one‐way ANOVA followed by Duncan‘s multiple range test, *p* < 0.05; Figure 4A,B). This RGP‐to‐RGI conversion phenomenon is different from the flg22‐triggered RGI observed in *Arabidopsis* [3, 24, 25, 26] and rice (Figure S10). In addition to microbial inocula compositions, the RGP‐to‐RGI conversions for NSM function in both mutants indicate potential links between immune perception and microbiome functionality.

**Figure 5:**
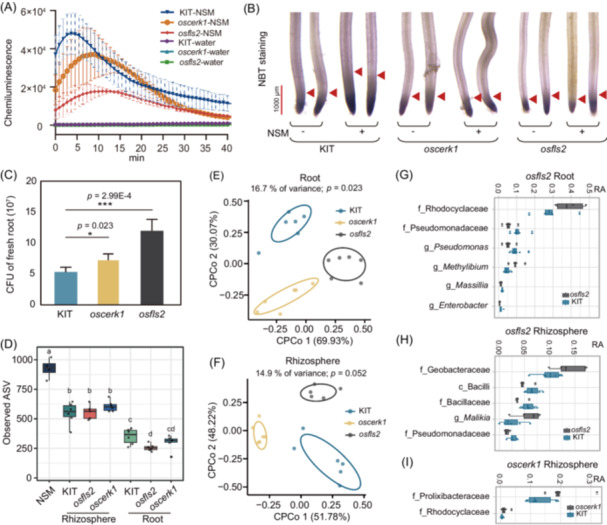
Microbiota dysbiosis in *osfls2* and *oscerk1* mutants. (A) Comparisons of NSM‐induced root ROS bursts among KIT, *osfls2*, and *oscerk1*. Data are presented as mean ± SD (*n* = 6 − 17). (B) NBT staining showing O_2_^−^ accumulation in the root tips at 14 days post‐NSM inoculation. Red arrows indicate positions with the same NBT staining intensity. Bar = 1 mm. (C) Comparisons of bacterial load (cfu g^−^^1^ fresh weight root) in the roots of the KIT, *osfls2*, and *oscerk1* roots. The samples were collected at 14 days post‐NSM inoculation. Data are presented as mean ± SD; *p < 0.05; ***p < 0.001 (Student's *t*‐test, *n* = 11−18). (D) Comparison of the bacterial richness (observed ASVs) of the input, rhizosphere, and root communities from the KIT, *oscerk1* and *osfls2* plants. Different letters above the boxes indicate significant differences between experimental variants, as determined by the Kruskal‐Wallis test followed by the Mann–Whitney *U* test. (E, F) Constrained PCoA (CPCoA) showing that the microbiotas of the KIT, *oscerk1* and *osfls2* plants separated from each other in the roots and rhizosphere samples. The variances explained by each principal coordinate axis are shown in brackets. (G−I) Relative abundance (RA) of enriched and depleted bacterial taxa in the rhizosphere and roots of the *osfls2* and *oscerk1* mutants. All *p*‐value < 0.05 (Kruskal–Wallis test).

We then investigated the host immune responses after inoculating NSM into wild‐type, *osfls2* and *oscerk1* roots. In KIT roots, NSM inoculation triggered reactive oxygen species (ROS) burst, a typical event of MTI responses (Figure 5A). In contrast, the amplitude of ROS burst was significantly reduced in *oscerk1* and *osfls2* roots, while *osfls2* displayed a more pronounced reduction of ROS burst. Nitro blue tetrazolium (NBT) staining revealed that O_2_^−^ signals in the roots of NSM‐inoculated KIT plants were stronger than those in the roots of germ‐free plants; however, NSM did not induce O_2_^−^ signals in the roots of *osfls2* and *oscerk1* (Figure 5B). Consistent with the compromised immunity in *osfls2* and *oscerk1* mutants, the number of culturable bacteria in the *osfls2* and *oscerk1* roots increased by 2.2‐ and 1.3‐fold, respectively, after NSM was inoculated in gnotobiotic bottles (Figure 5C). However, we found that NSM inoculation suppressed the expression of defense related genes, including five pathogenesis‐related (*PR*) genes and 3 WRKY transcription factors, which were induced by pathogen infection (Figure S11). These data imply that *osfls2* and *oscerk1* mutants compromised the immune responses but differentially regulated defense‐related genes.

Host immunity deficiencies disrupt microbiome homeostasis, typically manifesting as pathobiont expansion, reduced diversity, and depletion of beneficial taxa [45]. 16S rRNA gene‐based amplicon sequencing revealed that the α‐diversities of the rhizosphere and root communities of *osfls2* and *oscerk1* were similar to those of KIT, except for the *osfls2* root community, which had less bacterial richness (Figure 5D; ANOVA followed by post hoc Tukey's HSD test, *p* < 0.05). Constrained PCoA for genotype revealed that the bacterial communities of *osfls2* and *oscerk1* were compositionally separated from KIT, explaining 16.7% and 14.9% of the variation in the root and rhizosphere communities, respectively (Figure 5E,F). LEfSe (Linear discriminant analysis Effect Size) [46] analysis indicated that the *osfls2* root community had fewer Pseudomonaceae, *Enterobacter* and *Massilia* but more Rhodocyclaceae and *Methylibium*; while the *osfls2* rhizosphere community had fewer Bacillaceae and Pseudomonadaceae but more Geobacteraceae (Figure 5G,H and Table S3). The *oscerk1* rhizosphere communities had more Prolixibacteraceae and Rhodocyclaceae (Figure 5I). Together, alterations in the bacterial diversities of the *osfls2* and *oscerk1* microbiomes suggest that these two genes are required to maintain microbiome homeostasis.

### *OsFLS2* plays a prominent role in microbiota‐mediated regulation of host gene expression

**Figure 6:**
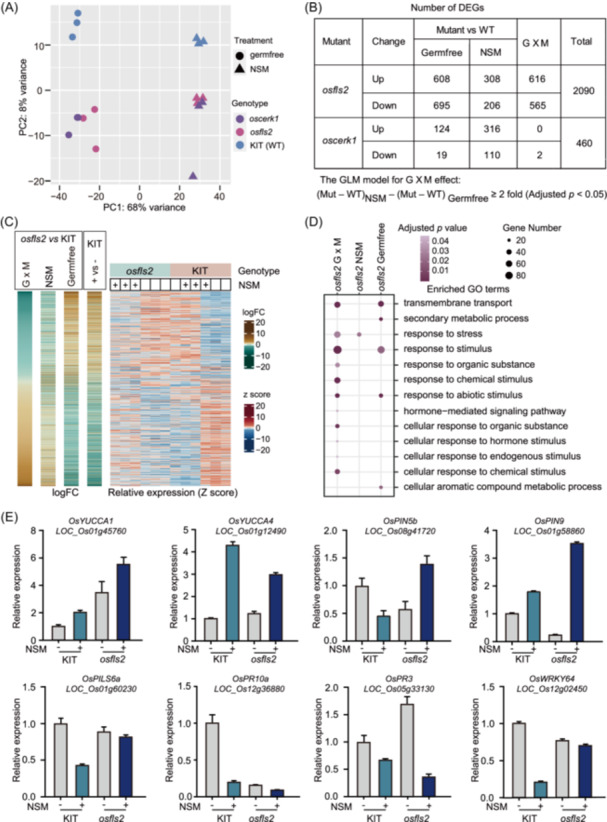
*osfls2* and *oscerk1* reprogram the expression of NSM‐responsive genes in roots. (A) Principal component analysis of the root transcriptomes of the WT (cv. KIT), *oscerk1* and *osfls2* mutant plants in germfree and NSM‐inoculated soils. (B) Number of DEGs associated with *oscerk1* and *osfls2* in germ‐free, NSM‐inoculated, and G × M conditions. DEGs were analyzed via a generalized linear model (GLM), and the model used to analyze genes associated to G × M is shown at the bottom. Mut, expression value in *osfls2* or *oscerk1* mutants; WT, expression value in wild‐type plants. (C) Heatmap showing the fold changes and normalized expression of *osfls2*‐associated DEGs. logFC, log_2_‐fold change; + vs −, fold changes of genes after NSM inoculation in wild type (KIT); *z* score, median‐centered *Z* score of expression value. (D) Comparison of enriched GO terms of the 2090 DEGs associated with *osfls2* in germ‐free, NSM‐inoculated, and G × M conditions. (E) The relative expression of auxin and defense‐related genes in the root samples was determined via RT‐qPCR. Data are presented as mean ± SD (*n* = 3 technical replicates). + and – indicate plants inoculated with live and filter‐sterilized NSM, respectively.

To further investigate host genes that contributed to RGP‐to‐RGI shift of NSM on *osfls2* and *oscerk1* mutants, we profiled the transcriptomes of KIT (wildtype), *osfls2* and *oscerk1* roots in germ‐free and NSM‐inoculated soils. Notably, 4534 genes were changed more than twofold in KIT after NSM inoculation, accounting for approximately 17% of the genes expressed in rice roots (Figure S12 and Table S4). Principal coordinate analysis revealed that these transcriptomes of *osfls2* and *oscerk1* were completely separated from WT in both germfree and NSM conditions (Figure 6A). We used a generalized linear mixed model in edgeR [47] to analyze differentially expressed genes (DEGs) associated with genotypes (G), microbial conditions (M), and their interactions (G × M). A total of 2090 and 460 DEGs were identified in *osfls2* and *oscerk1*, respectively (Figure 6B and Table S4; fold change ≥ 2, adjusted *p* < 0.05). Among them, 1181 (*osfls2*) and 2 (*oscerk1*) DEGs were associated with G × M, implying that many of the *OsFLS2*‐regulated genes were dependent on microbial conditions (Figure 6B). This large proportion of DEGs associated with the *osfls2* mutant and microbiota treatment suggests that *OsFLS2* plays a prominent role in NSM‐mediated regulation of host genes.

We next investigated the DEGs in *osfls2* mutants. Interestingly, 53% (1110/2090) of the *OsFLS2*‐regulated genes exhibited opposite fold changes between germ‐free and NSM‐inoculated conditions, whereas 89% (988/1110) of these genes transcriptionally responded to NSM inoculation in KIT (Figure 6C). A similar trend was also found in *oscerk1* DEGs (Figure S13). Interestingly, the functions of the *OsFLS2* DEGs were associated with cellular responses to various stimuli, including organic substances, chemical stimuli, and hormones (Figure 6D), which is similar to the enriched GO terms in NSM‐responsive genes in KIT (Figure S12). Furthermore, NSM‐mediated suppression of *OsPR10a, OsPR3*, and *OsWRKY64* were compromised in *osfls2* (Figure 6E). In agreement with the role of auxin in root growth and the rhizosphere microbiota [3, 24], the expression of at least 3 auxin efflux genes (*OsPIN5b*, *OsPIN9*, and *OsPILS6*) and 2 biosynthesis genes (*OsYUCCA1* and *OsYUCCA4*) was regulated by G × M in the *osfls2* transcriptome data, as further demonstrated by RT‐qPCR (Figure 6E), implying that the auxin pathway is involved in NSM‐mediated regulation of rice root growth. Together, these data show that the microbiota has profound effects on the *OsFLS2‐*mediated regulation of gene expression and, vice versa, that the knockout of these genes impairs the outcomes of rice‐microbiota interactions.

## DISCUSSION

**Figure 7:**
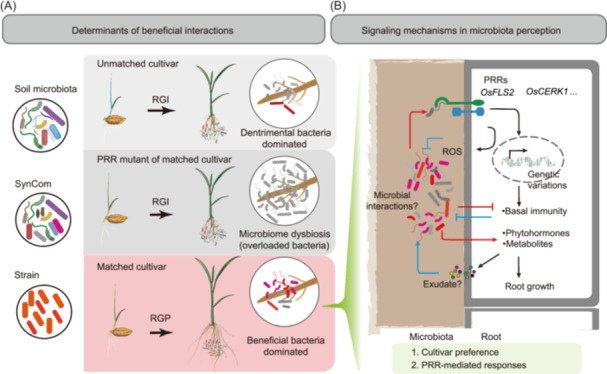
A working model of the two‐tiered regulatory framework governing rice–microbiota interactions. (A) Schematic representation of the phenotypic outcomes resulting from microbial inoculation. The native soil microbiota, synthetic communities, or individual bacterial strains exert beneficial (RGP), neutral, or detrimental (RGI) effects on root growth. These outcomes are determined by a two‐tiered regulatory system: the first tier is defined by cultivar‐specific microbial preference, and the second by host immune perception. Cultivar preference, shaped by host genetic variation, serves as the primary filter for microbial effects. PRR signaling, mediated by conserved receptor‐like kinases such as *OsFLS2*, constitutes the second tier. (B) A working model for microbiota‐modulated signaling in rice root. PRR activation by bacterial ligands reprograms host gene expression to fine‐tune phytohormone signaling, metabolic activity, and basal immunity. Dysregulation of this process, due to either cultivar mismatch or defective PRR signaling, shifts the interaction outcomes from RGP to RGI.

Reductionist approaches using gnotobiotic cultivation and SynCom have proven effective for deciphering microbiota assembly and function [48, 49, 50]. Applying a tailored gnotobiotic cultivation system (Figure 1 and Figure S1), we found that the NSM and SynCom promoted root growth in some cultivars, the effect of microbial inoculants shifted from RGP to RGI in a cultivar‐specific manner (Figures 1 and 3). This shift is associated with an imbalance in beneficial versus detrimental bacterial loads in roots. Furthermore, host immunity mediated by PRRs, particularly *OsFLS2*, acts as a critical gatekeeper of microbial homeostasis and the host transcriptional responses (Figures 4, 5, 6). We thus propose that microbiota effects are determined by a two‐tiered mechanism involving cultivar preference of microbiome and host immune perception (Figure 7).

### Microbiota‐cultivar compatibility is a key determinant of RGP

We propose that cultivar‐specific microbiota preference serves as a primary determinant in establishing beneficial crop‐microbe interactions (Figure 7A). In this framework, cultivar‐matched bioinoculants establish a microbiome dominated by beneficial bacteria, leading to RGP, whereas a mismatch between cultivar and bioinoculant results in RGI. This demonstration of cultivar‐level specificity within a single plant species extends the previously documented concept of host‐microbiota matching across different plant species (e.g., *Arabidopsis* and *Lotus japonicus* [51, 52]). These findings may facilitate the rational deployment of microbial inoculants in field applications [9]. This cultivar preference likely reflects implicit co‐selection during crop domestication, consistent with reported associations between host genetics and microbiome structures—such as the influence of *OsNRT1.1B* variation on the rhizosphere microbiome [13] or genetic variations impacting keystone microbes in maize [16]. Given the enrichment of metabolic functions among NSM‐responsive rice genes (Figure S12) and previously documented microbial substrate preferences [53, 54], we hypothesize that crop genetic variations among cultivars drive divergence in root exudates profiles [55], consequently influencing the colonization dynamics of specific bacterial taxa (Figure 7B). As a defined microbial system, SynCom11 provides a framework to dissect the role of bacterial functional traits in cultivar‐specific RGP by comparing matched versus unmatched cultivar‐SynCom combinations. Future studies integrating genomic and exometabolomic analyses of matched versus unmatched rice cultivar‐SynCom combinations will help to uncover the mechanistic basis of cultivar preference.

### Host PRRs are required for microbiome homeostasis in beneficial rice–microbiota interactions

The conversion from RGP to RGI in *osfls2* and *oscerk1* mutants suggested that host immunity, mediated by PRRs, acts as a crucial gatekeeper determining the outcomes of rice‐microbiota interactions (Figure 7A). Consistent with emerging roles of RLKs in maintaining microbiota homeostasis [22], these mutants exhibited increased bacterial loads and altered community structures (Figure 5), which are symptoms of microbial dysbiosis and dampened host health and growth [56]. Furthermore, our transcriptomic data reveal that *OsFLS2* is required for NSM‐mediated suppression of defense‐related genes (Figures 6C−E and Figure S11). This phenomenon is similar to observations that commensal and symbiotic bacteria suppress MTI in *Arabidopsis* [26, 57]. We therefore speculate that cultivar‐matched microbiota (or strains) was further controlled by host immunity through PRR‐mediated perception of microbial signals, which fine‐tunes immune responses to prevent microbiome dysbiosis.

The signaling pathways of this immune fine‐tuning (Figure 7B) appears distinct from canonical pathogen responses. Although soil microbiota activated the accumulation of ROS (Figure 5A,B), it concurrently suppressed the expression of defense‐related genes like PR genes in roots (Figures 6E and Figure S11). This bifurcated immune response—activating early signaling, while repressing late outputs—suggests that beneficial microbiota may intercept defense gene activation without impairing MAMP‐triggered ROS accumulation (Figure 7B). This hypothesis aligns with the established MTI signaling framework, where MAMP perception triggers ROS production via the phosphorylation of membrane‐anchored respiratory burst oxidase homologs, while activates PR gene expression via the phosphorylation of transcription factors [58]. Beyond immune signaling, the bacteria may also communicate with plant cells through diverse chemicals, including root exudates, phytohormones, volatile compounds, and microbial metabolites. Further dissecting regulatory networks that integrate host immunity, plant growth, and microbial chemical signaling will provide crucial insights into plant‐microbiota signaling pathways mediated by PRRs.

### Bacterial loads of beneficial taxa are critical determinants for microbiome functionality

The functional relevance of quantitative microbiome profiling is now evident across systems, from the mammalian gut [37, 38, 59] to plant root compartments [60]. In plants, changes in microbial load have been established as meaningful indicators of microbiome status under various conditions, including drought stress in rice, root rot disease in wheat, and different developmental stages in soybean [61, 62, 63]. Our study advances this understanding by demonstrating that the absolute load of beneficial bacteria, rather than 16S rRNA gene‐based relative abundance, serves as a reliable predictor of cultivar‐specific outcomes following SynCom11 inoculation (Figure 3H,I). Interestingly, the conversion from RGP to RGI observed in *osfls2* and *oscerk1* mutants correlates with significantly increased bacterial loads (Figure 5C), supporting a model in which host immunity actively constrains over proliferation of bacteria in roots (Figure 7A). These findings position absolute bacterial load as a decisive parameter for assessing microbiome functionality, which is dynamically tuned through the integration of host immunity, root exudate composition [54], and microbial interactions [62] (Figure 7B).

### Limitations of the study and future directions

While this study reveals that cultivar preference and host immune perception gate the establishment of a beneficial root microbiome, several key questions await future investigation. First, it is unclear whether natural variations in basal disease resistance among rice cultivars contribute to microbiota preference. Second, the role of chemical communication—such as that mediated by root exudates and microbial metabolites—in shaping beneficial microbial assemblies remains unexplored. Third, elucidating the complete signaling pathway from microbial ligand perception by PRRs to transcriptional reprogramming would identify valuable targets for applying genetic tools for microbiome engineering. Finally, general principles for designing cultivar‐matched SynComs remain to be established. These principles could integrate knowledge of ecological processes in microbiome assembly (Figure 2F) with molecular signaling pathways (Figure 7B). Addressing these challenges in future work will be essential to deepen the mechanistic understanding and enable rational design of host‐adapted microbiomes in crops.

## CONCLUSION

In conclusion, this study revealed that the cultivar preference of microbiota and host immune RLK receptors control the outcomes of rice–microbiota interactions (Figure 7). These findings emphasize the importance of cultivar‐matched microbiota and host immune response to achieve root‐growth‐promoting outcomes in rice–microbiota interactions.

## METHODS

### Preparation of gnotobiotic bottles for rice growth

The schematic design of the rice gnotobiotic is shown in Figure 1A and Figure S1. The first version (version 1) of the gnotobiotic culture system consisted of two sterilized and transparent polypropylene cups (height: 17.6 cm; diameter: 5.5 cm). A polytetrafluoroethylene (PTFE) membrane (diameter: 3 cm; pore size < 0.45 μm), which is used in tissue culture bottles, was inserted in the middle of the upper cup and used as a lid. This membrane allows air exchange but prevents airborne microorganisms. A gnotobiotic bottle is assembled by top‐to‐top stacking of an upper lid and a bottom cup, whereas the junction of two cups is sealed with parafilm or Saran wrap. This gnotobiotic bottle creates an axenic environment for rice growth and is suitable for rice morphology. To better mimic paddy field conditions, we designed an updated version of the gnotobiotic system (version 2) by incorporating a Whatman Nuclepore Track‐Etched membrane (pore size 0.03 μm, Cat # WHA800307) and a sponge at the bottom. This bottom filter membrane allows the diffusion of water and nutrients and is successfully applied for in situ bacterial culturation [64]. To prepare the upper lids and bottom cups, the cups were punched; then the membranes were glued using silicone aquarium sealant (e.g., Kafuter product code K704). The assembled version 2 gnotobiotic bottle can be placed in aquatic solution and paddy fields, allowing the exchange of nutrients and metabolites between inside and outside soils (Figure S1A−D).

For rice cultivation, the cups were sterilized with ultraviolet light irradiation and assembled in a germ‐free hood. For each gnotobiotic bottle, 40 g of autoclaved soil and 50 mL of water (or nutrient solution) were placed in the bottom cup. This gnotobiotic bottle could be opened and reassembled in a tissue culture hood to perform various experiments.

### Preparation of soil for gnotobiotic bottle growth systems

Paddy field soils were collected in the campus of Huazhong Agricultural University, Hubei province (paddy soil #1 and #2) and in a farm of XingAn, Inner Mongolia Autonomous Region (paddy soil #3), passed through a 2‐mm sieve to remove large particles, homogenized, and stored at room temperature until use. Peat soil (equivalent to peat potting mix and medium vermiculite substrate that is commonly used for *Arabidopsis* growth) was prepared by mixing Pindstrup substrate and loam in a 1:1 ratio. The physical and chemical properties of the soils were measured by the United Nations Quality Detection Group Co., Ltd. (Xi'an, China) (Figures S2A and 2B). For soil sterilization, 40 g of soil was moistened, placed in 1‐L aluminum boxes (21 cm × 12 cm × 5.5 cm, L × W × H) and autoclaved three times at 121°C for 60 min. Each gnotobiotic bottle contained 40 g of autoclaved soil and 50 mL of autoclaved H_2_O, which maintained water flooding condition as the paddy field. Unless otherwise specified, paddy soil #2 was used throughout this study. The exceptions are the experiments presented in Figure 1G,H. Before the rice seeds were planted, a small aliquot of soil from gnotobiotic bottles was plated on 0.5 × tryptic soy agar (TSA) plates (Solarbio) to check for contamination, and the contaminant bottles were discarded. Germinated rice seeds were placed on soil in gnotobiotic bottles and grown in a growth chamber. In all the experiments, four sterile rice plants were grown in one gnotobiotic bottle.

### Preparation and inoculation of NSM

For NSM preparation, 200 g of mud bulk soil was collected from a paddy field planted with different rice cultivars for at least 10 years on the campus of Huazhong Agriculture University (30.474377° N, 114.356026° E) and thoroughly mixed with 1 L of autoclaved water. After the particles were removed by sedimentation, the upper layer soil slurry was used as the NSM. The number of culturable bacteria in the NSMs used in this study was approximately 5.0–6.8 × 10^6^ CFU/g fresh paddy soil, which was counted via 0.1 × TSA plates. For each gnotobiotic bottle, 4 mL of NSM was added to the soil in a germ‐free hood. An NSM solution sterilized by passage through a 0.22‐µm membrane filter (Millipore) or autoclaved (heat‐killed) was used as the control.

### Bacterial isolation

To isolate rice‐associated bacteria, rice roots were collected from paddy fields, homogenized and suspended in autoclaved MgCl_2_ solution (10 mM). The suspensions were serially diluted and spread on 0.1 × TSA plates (Solarbio). Individual colonies were selected and cultured in 0.1 × tryptic soy broth (TSB). A total of 1629 bacterial isolates were picked via this spread‐up approach. For high‐throughput cultivation of bacteria from rice root suspensions, we followed a previously described protocol [36] and obtained 5166 bacterial isolates. All bacterial isolates were stored in 25% glycerol at −80^°^C. The 16S rRNA genes of each bacterial isolate were amplified via 799 F and 1193 R primers, and the amplicons were indexed, pooled, and sequenced with an Illumina HiSeq. 2500 platform (Shanghai Personal Biotechnology Co., Ltd.). The amplicon reads were quality‐filtered, demultiplexed, denoised, merged, and then used for taxonomic assignment of bacterial isolates via QIIME 2 [65] and the Greengenes database (https://greengenes2.ucsd.edu).

### Inoculation of SynComs and individual strains

The design of SynCom is depicted in Supplementary Note 2 and Figure S5. For SynCom or single‐strain inoculants, bacterial stocks were inoculated into 0.1 × TSB media and cultured at 28°C for 24–36 h. The bacterial cells were subsequently collected via centrifugation and washed with sterilized water. The bacterial cells were suspended to a final concentration of OD_600_ = 0.5. SynComs were prepared by mixing equal volumes of each strain [13]. For each gnotobiotic bottle, 0.15 mL of bacterial inoculant was added. Rice plants inoculated with the same volume of autoclaved bacterial mixture (heat‐killed) were used as control groups.

### Binary bacterial inhibition assay

Bacterial interaction assays were performed as described by Chen et al. [22]. Briefly, 3 ml of a “target” bacterial suspension (OD_600_ = 2.0) was mixed with 15 mL of molten 0.5 × TSB agar‐based medium at 42°C and then poured into square Petri dishes (90 × 90 mm). Then, 3 μL of each “Attacker” strain (OD_600_ = 2.0) was spotted onto the plates, with three replicates. The bacteria on the plates were cultured at 28°C for 3–5 days, after which photographs were taken to observe the inhibition halos. The experiments were conducted twice.

### Rice cultivars and growth conditions

Seeds of 10 different cultivars were obtained from the rice germplasm collection in Professor Lizhong Xiong's laboratory at Huazhong Agricultural University. The rice mutants *osfls2* (LOC_Os04g52780) and *oscerk1* (LOC_Os08g42580) were obtained from the CRISPR/Cas9 genome editing library of rice RLK/RLCK genes [40].

For the preparation of sterilized rice plants, the dehulled rice seeds were washed two times with 75% alcohol, soaked in 0.1% HgCl_2_ for 6 min, and subsequently washed 5 times with sterilized H_2_O. The surface‐sterilized rice seeds were germinated on 0.5 × Murashige and Skoog (MS) media supplemented with 0.25% Phytagel (PhytoTech). Germinated rice seeds without bacterial contamination were transplanted into autoclaved soil in gnotobiotic bottles. Rice plants were grown at 28°C with 16 h of light and 8 h of darkness.

For the rice–microbe interaction assays in agar media (Figure 1B), sterilized and geminated rice seeds were placed in glass tubes containing 50 mL of 0.5 × MS media supplemented with 0.25% Phytagel (PhytoTech). For hydroponic cultivation, sterilized and germinated rice seeds were grown in sterilized Hoagland's nutrient solution (Coolaber).

### Root phenotyping

Roots inoculated with microorganisms for 14 days were removed from the gnotobiotic growth bottles and carefully washed with water to remove soil particles. The roots were scanned and measured via WinRHIZO (LA2400 Scanner). For cross‐sectional images, root tips (~3 cm from the rice root tip), comprising the root cap, meristematic, elongation, and differentiation zones, were embedded in 3.5% melted agarose [66]. Transverse sections (40 µm thick) were cut using a Leica VT 1200S vibratome and imaged with a Nikon MODEL ECLIPSE Ni‐E microscope.

### Sample collection and preparation for 16S rRNA gene sequencing

Rhizosphere and root samples were collected as described previously [67]. NSM inoculates were prepared as described above, and bacteria in 4 ml of NSM solution (equal volume of NSM inoculants) were collected by centrifugation and stored at −80°C. Then, 4 mL of NSM was inoculated into rice seedlings growing in organic soil and loam in gnotobiotic bottles. After 14 days, the rice roots were collected and washed two‐times with sterilized 1× PBS (pH = 7.2) and stored at −80°C. After the roots were removed, rhizospheric soil samples were collected from the gnotobiotic bottles. To analyze the microbiome of MH63 rice, 6 biological replicates were used, and a total of 30 samples were analyzed. For the KIT, *osfls2* and *oscerk1* experiments (Figure 5), six biological replicates were used, and a total of 42 samples were analyzed.

For 16S rRNA gene amplicon sequencing, the genomic DNA of these samples was extracted via DNeasy PowerSoil Pro Kits (QIAGEN) following the manufacturer's instructions. Briefly, 16S rRNA gene amplicons were prepared via a two‐step PCR procedure as we described previously, which depleted host organelle rRNA fractions through CRISPR/Cas9 cleavage of first‐round PCR products [67]. The barcoded amplicons were pooled and subjected to 2 × 250 paired‐end sequencing with an Illumina HiSeq. 2500 platform. For each PCR step, negative controls (using H_2_O as a template) were always included to ensure that the reagents and laboratory consumables were not contaminated by bacteria.

### Analysis of 16S rRNA gene amplicons

The 16S rRNA gene sequences were processed via the DADA2 pipeline [68]. Briefly, adapter sequences were trimmed, and low‐quality reads were filtered from the raw sequencing reads. Here, the forward and reverse reads were trimmed to 210 bp, and a maximum of two expected errors were allowed. ASVs were generated from clean reads via DADA2 software [68]. Taxonomy assignment was conducted via DADA's assignTaxonomy function in the Silva 132.1 reference database [69]. ASVs assigned to the mitochondrial, chloroplast, or nonbacterial kingdom and low‐abundance sequences (<20 in all samples) were discarded. The criteria for defining core ASVs are ASV relative abundance > 0.01 and prevalence = 100%. Diversity analysis was performed via the phyloseq. 1.38.0 [70] and vegan R packages 2.6‐4 [71] via ASV tables from rarefied datasets. Analysis of the differential taxa of the WT and *osfls2* and *oscerk1* mutants was carried out via LEfSe [46] with LDA significance threshold >2.

The iCAMP analysis was performed using the R package “iCAMP” (version 1.5.12) [33], and the ecological stochasticity was quantified as the normalized stochasticity ratio based on phylogenetic metrics (pNST) using R package “NST” (version 3.1.10) [72]. Unless otherwise specified, parameters are set to default values. Briefly, a rooted phylogenetic tree was first constructed from the ASV table using the FastTree algorithm in QIIME2 (2020.11). The weighted βNTI and RCbray indices were calculated using the pNST function with 999 random permutations. Deterministic and stochastic processes were inferred from |βNTI| and |RCbray| values according to the established framework [33]. Deterministic processes were identified when |βNTI| > 2, while stochastic processes were determined when |βNTI| < 2. Furthermore, |RCbray| > 0.95 was used to distinguish between dispersal limitation (RCbray > 0.95) and homogenizing dispersal (RCbray < −0.95), whereas |RCbray| < 0.95 indicated drift alone.

### Bacterial load quantification via qPCR and plate count

To assess the bacterial load of each strain, roots inoculated with SynCom11 for 14 days were collected from gnotobiotic growth system and washed with water to remove the soil. The DNA from root tissues was extracted using DNeasy PowerSoil Pro Kits (QIAGEN), and 5 ng DNA as template in 10 μL reaction volumes containing TB Green Premix Ex Taq II (Tli RNaseH Plus, Takara) and gene‐specific primers. The amplification efficiencies of all primer pairs were examined by LinRegPCR [73] and primer‐pairs with amplification efficiencies higher than 1.95 were used. The bacterial gene copy number (bacterial load in root) was normalized to rice *UBIQUITIN10* (*OsUBQ10*) and calculated as follows: bacterial gene/plant gene = 2^Ct (plant gene)^/2^Ct (bacterial gene)^, where Ct (Cycle number to threshold) is the mean of the triplicate PCR reactions. The gene‐specific primers used in this study are listed in Table S5.

To enumerate culturable bacteria, root and soil samples were weighed, homogenized, and transferred to tubes. Each sample was then suspended in sterile distilled water. Serial dilutions of the suspensions were prepared and spread onto 0.1 × TSA plates (Solarbio) and incubated 28°C. Colonies were counted after 3 days of incubation.

### NBT staining and ROS burst assays

NBT was used to determine the O_2_^−^ concentration in the rice roots [74] at 14 days post‐NSM inoculation. For NBT staining, the rice roots were immersed in a 50 mL tube containing 1 × PBS (pH 7.8) supplemented with 0.1% (w/v) NBT and stained for 40 min. Then, the roots were placed in ClearSee solution (chloral hydrate:H_2_O:glycerol (m:v:v) = 8:3:1) for 24 h. The stained roots were imaged with a Nikon SMZ25 stereomicroscope (Nikon).

The ROS burst assays were performed as described previously with modifications [75]. Briefly, 5‐mm‐long root tip segments were excised from germ‐free rice seedlings (14 days post‐germination) growing on 0.5 × MS agar (with sugar) media. The rice segments were placed in a 96‐well microtiter plate containing 60 µL of 1 × PBS and incubated overnight at room temperature in the dark. Then, 40 µL of the elicitation solution (40 µg/mL horseradish peroxidase, 60 µM L‐012, and microbiota elicitation) was added to each well, and the luminescence was monitored every 1 min for a 120‐min period via a TECAN Spark microplate reader with a signal integration time of 0.5 s. Here, the microbial elicitation was prepared by mixing 5 mL of freshly prepared NSM and 5 mL of water.

### Flg22‐treatment of rice seedling

The surface‐sterilized rice seeds (KIT and *osfls2*) were germinated on 0.5 × MS media (without sugar) supplemented with 0.25% Phytagel (PhytoTech). After incubating at 28°C for 5 days, germinated seeds without bacterial contamination were transplanted into 0.5 × MS media (without sugar) supplemental with 0 and 1 µM flg22 peptide in plates. The rice plants were tilted for 1 week in a growth chamber at 28°C with 16 h of light and 8 h of darkness. The flg22 peptide (QRLSTGSRINSAKDDAAGLQIA), synthesized by Chenghengqian Biotechnology (Wuhan, China), was dissolved in distilled water and filter‐sterilized through a 0.22 μm membrane. The root phenotype was examined at 7 days post flg22 treatment.

### RNA‐seq analysis

Rice roots inoculated with NSM were collected from the gnotobiotic growth system at 14 days post inoculation and washed with water to remove the soil. Total RNA was extracted via TRIzol Reagent (Invitrogen). The RNA‐seq library was prepared via an NEBNext Ultra RNA Library Prep Kit (NEB) and sequenced via an Illumina NovaSeq 6000 (Novogene Co., Ltd.). An average of 6 million clean reads per sample was generated. The RNA‐seq reads were subsequently mapped to the *Oryza sativa japonica* cv. Nipponbare reference genome (GCF_001433935.1) and quantified via Salmon [76] with the default settings. Gene expression values were imported via the tximport R package 1.22.0 [77]. Differential gene expression analysis was performed via DESeq2 1.34.0 [78] and edgeR 3.36.0 [79]. DEGs were selected with the thresholds |log_2_FoldChange| ≥ 1 and Benjamini–Hochberg adjusted *p*‐value < 0.05. GO enrichment and KEGG enrichment analyses of the DEGs were performed with TBtools 1.6 [80]. For the RNA‐seq data of *osfls2* and *oscerk1*, the effects of genotype (mutant vs. WT), microbiota (NSM‐inoculated vs. germ‐free), and their interactions on gene expression were tested via the generalized linear model and quasi‐F test in edgeR ( ~ G + M + G × M). The G × M effects (fold change) were calculated via the following design model: (mutant – WT)_NSM_ – (mutant – WT)_Germfree_. The DEGs were selected with the thresholds |log_2_FoldChange| ≥ 1 and an adjusted *p*‐value < 0.05.

### RT‐qPCR

Total RNA from the root tissues was extracted using the TRIzol method and treated with DNase I (TaKaRa) to remove residual genomic DNA contaminants. Approximately 2 μg of total RNA was used for cDNA synthesis with oligo(dT)_18_ primers and Moloney murine leukemia virus (M‐MLV) reverse transcriptase (Takara). Real‐time PCR was performed using TB Green Premix Ex Taq II (Tli RNaseH Plus, Takara) and gene‐specific primers. Here, *OsUBQ10* was used as the reference gene. The relative expression level of each gene was analyzed via the delta‐delta Ct method.

### Statistical analysis

The R package (version 4.3.2), GraphPad Prism 9.0 and Microsoft Office Excel 365 were used for statistical analysis. Significant differences between two groups were analyzed by using two‐tailed student's *t*‐tests. Significant differences between multiple samples were determined by one‐way analysis of variance (ANOVA) followed by Duncan's multiple range test or two‐way ANOVA followed by post hoc Tukey's HSD test.

## AUTHOR CONTRIBUTIONS

**Jiwei Xu**: Conceptualization; methodology; validation; data curation; writing—original draft; investigation; resources; visualization. **Peiyao Hu**: Methodology; software; data curation; validation; visualization. **Meng Liu**: Methodology; visualization; validation. **Wanyuan Zhang**: Resources. **Kabin Xie**: Conceptualization; writing—original draft; writing—review and editing; funding acquisition; visualization; project administration; supervision; methodology. All authors have read the final manuscript and approved it for publication.

## CONFLICT OF INTEREST STATEMENT

The authors declare no conflicts of interest.

## ETHICS STATEMENT

No animals or humans were involved in this study.

## Supporting information


**Figure S1:** The design and demonstration of rice gnotobiotic bottles.
**Figure S2:** Effects of NSM on the root growth of 10 rice cultivars.
**Figure S3:** Analysis of ASV rank‐abundance relationships for core microbiota definition.
**Figure S4:** Inferring the ecological processes of community assembly in gnotobiotic cultivation.
**Figure S5:** Schematic diagram depicting the bottom‐up design of SynCom22 and simplification from SynCom22 to SynCom11.
**Figure S6:** Effects of SynCom11 on root growth of 10 cultivars.
**Figure S7:** Binary inhibition assays between 11 strains on 0.1 × TSA plates.
**Figure S8:** Absolute and relative quantification of bacterial load in rice root.
**Figure S9:** Screening of 2 subgroups of RLKs involved in NSM‐mediated regulation of root growth.
**Figure S10:** *Osfls2* mutant impairs flg22‐triggered root growth inhibition.
**Figure S11:** Suppression of rice defense‐related genes by NSM inoculation.
**Figure S12:** Rice genes responding to NSM‐inoculation in KIT roots.
**Figure S13:** Heatmap showing the fold changes and normalized expression of *oscerk1*‐associated DEGs.

**Table S1:** ASV abundances and taxonomic annotation of the input NSM, rhizosphere and root samples of MH63 and KIT.
**Table S2:** Taxonomy and 16S rRNA sequences of bacteria in SynCom22 and SynCom11.
**Table S3:** Enriched and depleted bacterial taxa in *osfls2* and *oscerk1* mutants.
**Table S4:** Differentially expressed genes in KIT, *osfls2* and *oscerk1* roots at 14 days post‐NSM inoculation.
**Table S5:** DNA oligos used in this study.
**Table S6:** Strain selection criteria for the construction of SynCom11 from SynCom22.

## Data Availability

The sequencing data reported in this paper have been deposited in the Genome Sequence Archive in the BIG Data Center [81] under project PRJCA025585 (https://ngdc.cncb.ac.cn/search/specific?db=bioproject&q = PRJCA025585). The computer scripts for 16S rRNA amplicon data and RNA‐seq data processing were deposited in GitHub (https://github.com/peiyaohu/Xu_et_al_iMeta_2025). Supplementary materials (figures, tables, graphical abstract, slides, videos, Chinese translated version, and update materials) may be found in the online DOI or iMeta Science http://www.imeta.science/.
