# Supplementary material for: Cultivar‐specific preference of bacterial communities and host immune receptor kinase modulate the outcomes of rice–microbiota interactions
Source: Imeta. 2025 Dec 16;4(6):e70098. doi: 10.1002/imt2.70098 (PMC12747538; doi:10.1002/imt2.70098)
Supplement: Supplementary file 1 — Figure S1: The design and demonstration of rice gnotobiotic bottles. Figure S2: Effects of NSM on the root growth of 10 rice cultivars. Figure S3: Analysis of ASV rank‐abundance relationships for core microbiota definition. Figure S4: Inferring the ecological processes of community assembly in gnotobiotic cultivation. Figure S5: Schematic diagram depicting the bottom‐up design of SynCom22 and simplification from SynCom22 to SynCom11. Figure S6: Effects of SynCom11 on root growth of 10 cultivars. Figure S7: Binary inhibition assays between 11 strains on 0.1 × TSA plates. Figure S8: Absolute and relative quantification of bacterial load in rice root. Figure S9: Screening of 2 subgroups of RLKs involved in NSM‐mediated regulation of root growth. Figure S10: Osfls2 mutant impairs flg22‐triggered root growth inhibition. Figure S11: Suppression of rice defense‐related genes by NSM inoculation. Figure S12: Rice genes responding to NSM‐inoculation in KIT roots. Figure S13: Heatmap showing the fold changes and normalized expression of oscerk1‐associated DEGs. [file IMT2-4-e70098-s001.docx]

**Supporting information to**

**Cultivar-specific preference of bacterial communities and host immune receptor kinase modulate the outcomes of rice-microbiota interactions**

**Running title:** The gated-control of rice-microbiota interaction outcomes

Jiwei Xu^1, 2^, Peiyao Hu^1, 2^, Meng Liu^1, 2^, Wanyuan Zhang ^1, 2^, Kabin Xie^1, 2*^

^1^National Key Laboratory of Crop Genetic Improvement, Hubei Hongshan Laboratory, Huazhong Agricultural University, Wuhan 430070, China

^2^Hubei Key Laboratory of Plant Pathology, Huazhong Agricultural University, Wuhan 430070, China

*Correspondence: [kabinxie@mail.hzau.edu.cn](mailto:kabinxie@mail.hzau.edu.cn) (Kabin Xie)

**Supplementary Note 1: A Tailored Gnotobiotic Growth System for Modeling Rice-Microbiota Interaction Studies**

We aimed to investigate how different rice cultivars establish a beneficial microbiome from the soil microbiota and bioinoculants. Establishing causality between plant phenotype and microbiome diversity [1] requires gnotobiotic cultivation systems that can grow plants with defined and controlled microbiotas. Several gnotobiotic systems have been designed to investigate plant interactions with growth-promoting bacteria such as *Rhizobium* symbionts [2−5]. Recently, James designed FlowPot and GnotoPot to investigate microbiota*-Arabidopsis* interactions [6]. However, these devices are not suitable for rice because rice plants have distinct morphologies and growth conditions. Unlike *Arabidopsis* and many other crops, rice grows under semiaquatic conditions (anaerobic); therefore, rice-microbe interaction experiments using agar plates or nonflooded soil rarely emulate paddy field conditions. Hence, a gnotobiotic growth device that fits rice morphology (tall and slender) is needed to simulate semiaquatic paddy field conditions for rice-microbiota interaction studies.

We developed two versions of gnotobiotic cultivation systems to study rice-microbiota interactions. Version 1 provided a fully enclosed environment, while Version 2 permitted controlled nutrient exchange without microbial contamination (Figure S1A–D). To evaluate their performance, we inoculated germ-free MH63 rice seedlings in both systems with the same natural soil microbiota (NSM). The Version 2 assemblies were partially submerged in Hoagland's nutrient solution to enable solute exchange. After 14 days, both systems supported comparable NSM-mediated root growth promotion in MH63 (Figure S1G,H). However, the increase in shoot biomass was only observed in Version 2 following NSM inoculation (two-way ANOVA with Duncan’s post hoc test, *p* < 0.05; Figure S1H), highlighting the importance of nutrient availability in shaping microbiota‐dependent shoot growth.

For long-term experiments, the gnotobiotic bottles could be extended by stacking multiple bottom-removed cups in the middle (Figure S1I,J). For early flowering cultivars, such as Kitaake (KIT), rice plants exhibit normal flowering and seeding in gnotobiotic bottles, but tillering is inhibited in those plants, likely due to restricted space in the bottles.

In this study, we analyzed the phenotype, microbiome assemblages, and gene expression at 14-day post inoculations based on the following pilot experimental data: (1) Quantitative culturing revealed that the bacterial density (CFU/g) in the gnotobiotic system at 14 days post-inoculation reached a magnitude comparable to that of natural paddy soil (Figure S1F); (2) By this time point, root phenotypic differences between germ-free and microbiota-inoculated plants were clearly evident, and the shoots had reached the physical limit of the bottle height. Based on these observations, we determined that 14 days represented an appropriate timeframe to assess the establishment of microbiota and its effects on plant growth.

**Supplementary Note 2: The Design of a Simplified SynCom for Rice Gnotobiotic Cultivation**

Depending on the objectives and experimental systems, many strategies were used to design SynComs for animal and plant microbiome studies, which are summarized as a continuum of bottom-up to top-down designs [7,8]. The top-down designs employ host and environmental filtering or a sequential dropout of a large community, resulting in a less complex SynCom with desired traits. For instance, host-mediated selection of natural root microbiota resulted in a simplified SynCom consisting of seven strains in maize [9]. In the bottom-up design, bacterial strains, which represent phylogenetic diversity of the natural community at some level or have specific functions, are selected to assemble a community. The criteria of strain selection are crucial for bottom-up designs and the effectiveness of designed SynCom requires experimental evaluation. The following aspects are considered for strain selection: the representativeness of bacterial taxa in natural communities, the source of the strains, the ease of cultivation and growth rate, and the characterized or predicted function of strains. According to the diverse objectives of different studies, the selection criteria of bacterial strains are different. The size of the SynComs ranges from hundreds of strains [10,11], which maximally cover the diversity of natural soil communities, to several bacterial strains with specific functions (*e.g.*, growth promotion and disease suppression) [12,13].

Our aim is to assemble a bacterial consortium to mimic the RGP effects of NSM but have minimal complexity. According to these aims, we used the bottom-up approach to design the SynCom with the following considerations and procedures.

1) Covering the four dominant phyla of soil bacteria.

2) The purity of bacterial isolates obtained through high-throughput cultivation in 96-well plates was ensured by a stringent filtering step: only isolates exhibiting > 95% identity in their 16S rRNA gene sequences were retained. This criterion effectively excluded wells containing mixed strains and guaranteed the genetic homogeneity of each selected isolate.

3) Including the bacterial families in the core microbiota of rice roots, including Rhodocyclaceae, Burkholderiaceae, Pseudomonadaceae, and Bacillaceae.

4) Ease of cultivation. We selected strains that grow to saturation within 36 h in 0.1 × TSB.

Following these criteria, we obtained SynCom22 consisting of 22 strains from the four major bacterial phyla found in the rice roots and 13 distinct bacterial families (Figure S5).

SynCom11 was constructed from SynCom22 through a structured reduction process that halved the number of strains while preserving phylogenetic breadth. The following criteria guided the selection:

1) At least two strains but no more than four strains were retained from each of the four original phyla.

2) For bacteria within the same family, one strain was randomly selected and the others excluded.

3) Three bacterial families, namely Enterobacteriaceae, Exiguobacteraceae, and Sphingobacteriaceae, were deliberately omitted from SynCom11 to maintain the limit of no more than four strains per phylum.

The resulting simplified community SynCom11 maintained broad phylogenetic diversity at the phylum level. A detailed rationale for the exclusion of each strain is provided in Table S6.

**REFERENCES**

1. Vorholt, Julia A., Christine Vogel, Charlotte I. Carlström, Daniel B. Müller. 2017. “Establishing causality: opportunities of synthetic communities for plant microbiome research.” *Cell Host & Microbe* 22: 142-155. <https://doi.org/10.1016/j.chom.2017.07.004>

2. Leonard Lewis, T. 1943. “A simple assembly for use in the testing of cultures of rhizobia.” *Journal of Bacteriology* 45: 523-527. <https://doi.org/10.1128/jb.45.6.523-527.1943>

3. Rovira, A. D., G. D. Bowen. 1966. “The effects of micro-organisms upon plant growth.” *Plant and Soil* XXV: 129-142. <https://doi.org/10.1007/BF01347967>

4. Hale, M. G., D. L. Lindsey, K. M. Hameed. 1973. “Gnotobiotic culture of plants and related research.” *The Botanical Review* 39: 261-273. <https://doi.org/10.1007/BF02860119>

5. Kloepper, J. W. , M. N. Schroth. 1981. “Plant growth-promoting rhizobacteria and plant growth under gnotobiotic conditions.” *Phytopathology* 71: 642-644. <https://doi.org/10.1094/Phyto-71-642>

6. Kremer, James M., Reza Sohrabi, Bradley C. Paasch, David Rhodes, Caitlin Thireault, Paul Schulze-Lefert, James M. Tiedje, Sheng Yang He. 2021. “Peat-based gnotobiotic plant growth systems for *Arabidopsis* microbiome research.” *Nature Protocols* 16: 2450-2470. <https://doi.org/10.1038/s41596-021-00504-6>

7. Nrthen, Trent R., Manuel Kleiner, Marta Torres, Ákos T. Kovács, Mette Haubjerg Nicolaisen, Dorota M. Krzyżanowska, Shilpi Sharma, et al. 2024. “Community standards and future opportunities for synthetic communities in plant-microbiota research.” *Nature Microbiology* 9: 2774-2784. <https://doi.org/10.1038/s41564-024-01833-4>

8. Mehlferber, Elijah C., Gontran Arnault, Bishnu Joshi, Laila P. Partida-Martinez, Kathryn A. Patras, Marie Simonin, Britt Koskella. 2024. “A cross-systems primer for synthetic microbial communities.” *Nature Microbiology* 9: 2765-2773. <https://doi.org/10.1038/s41564-024-01827-2>

9. Niu, Ben, Joseph Nathaniel Paulson, Xiaoqi Zheng, Roberto Kolter. 2017. “Simplified and representative bacterial community of maize roots.” *Proceedings of the National Academy of Sciences* 114: E2450-E2459. <https://doi.org/10.1073/pnas.1616148114>

10. Pfeilmeier, Sebastian, Gabriella C. Petti, Miriam Bortfeld-Miller, Benjamin Daniel, Christopher M. Field, Shinichi Sunagawa, Julia A. Vorholt. 2021. “The plant NADPH oxidase RBOHD is required for microbiota homeostasis in leaves.” *Nature Microbiology* 6: 852-864. <https://doi.org/10.1038/s41564-021-00929-5>

11. Gonin, Mathieu, Isai Salas-González, David Gopaulchan, Juan P. Frene, Stijn Roden, Bram Van de Poel, David E. Salt, Gabriel Castrillo. 2023. “Plant microbiota controls an alternative root branching regulatory mechanism in plants.” *Proceedings of the National Academy of Sciences* 120: e2301054120. <https://doi.org/10.1073/pnas.2301054120>

12. Jin, Xue, Huiting Jia, Lingyi Ran, Fengzhi Wu, Junjie Liu, Klaus Schlaeppi, Francisco Dini-Andreote, Zhong Wei, Xingang Zhou. 2024. “Fusaric acid mediates the assembly of disease-suppressive rhizosphere microbiota via induced shifts in plant root exudates.” *Nature Communications* 15: 5125. <https://doi.org/10.1038/s41467-024-49218-9>

13. Gonçalves, Osiel S., Christopher J. Creevey, Mateus F. Santana. 2023. “Designing a synthetic microbial community through genome metabolic modeling to enhance plant-microbe interaction.” *Environmental Microbiome* 18: 81. <https://doi.org/10.1186/s40793-023-00536-3>


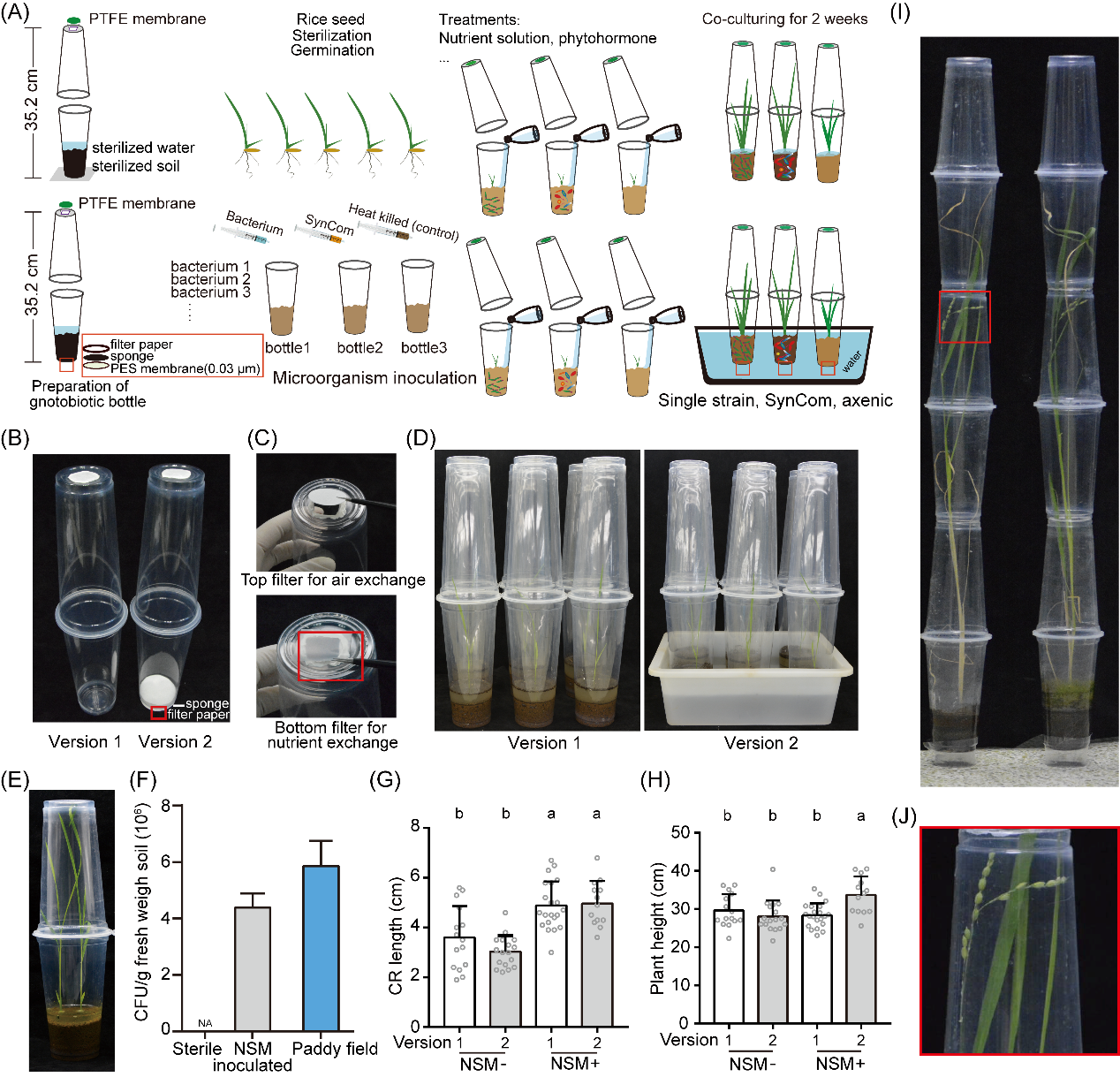


**Figure S1 The design and demonstration of rice gnotobiotic bottles.** (A) Workflow of the use of gnotobiotic bottles for rice growth. Two versions of gnotobiotic bottles were designed, whereas version 2 has a membrane in the bottom cup for the exchange of small molecules with the environment. A version 2 bottle can be put in solution or in a paddy field and permits the exchange of water and small molecules with the environment but prevents microbes from entering the gnotobiotic bottle. Germ-free rice seeds are planted in gnotobiotic bottles after germination on 0.5 × MS plates and inoculated with microorganisms or kept in germ-free conditions. (B and C) Photos showing the membranes in the upper lid and bottom cups. (D) Photos of rice plants growing in version 1 and version 2 bottles. Version 2 was placed in water to model rice-microbiota interactions in paddy fields. Nutrient solutions could be used to fill the tray to model rice-microbiota with different nutrient levels using gnotobiotic bottles. (E−J) Demonstrations of gnotobiotic bottles (version 2) for rice cultivation. The gnotobiotic bottles were placed in a tray filled with water. (E) A photo showing the rice plants in gnotobiotic bottles (version 2). (F) Examination of bacterial cells in gnotobiotic bottles. The bacteria in soil samples were counted after plating on 0.1 × tryptic soy agar (TSA) plates. NA, no colony was detected in gnotobiotic bottles without inoculating NSM. NSM inoculated, bacterial cfu in soil in gnotobiotic bottles inoculating NSM; paddy file, bacterial cfu from rice paddy field. cfu, colony-forming unit. (G and H) Comparison of 2 versions of gnotobiotic bottles. NSM had the same RGP effect on the rice plants growing in the two versions. However, plants in version 2 presented greater shoot length after NSM inoculation. (I and J) Rice (cv. KIT) plants showing normal flowering and seeding in sterilized gnotobiotic bottles. Tillering was inhibited due to space limitations in gnotobiotic bottles.


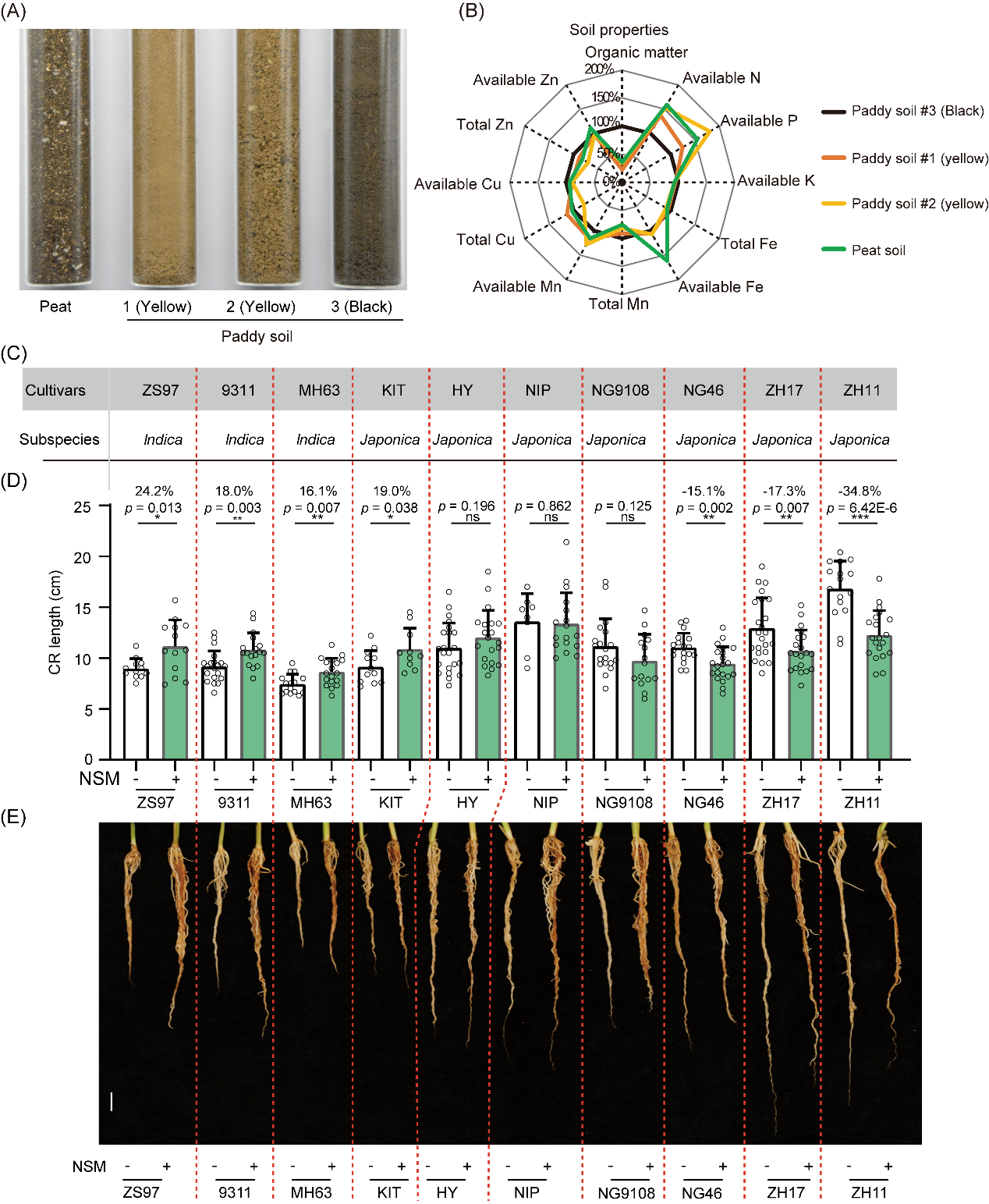


**Figure S2 Effects of NSM on the root growth of 10 rice cultivars.** Rice cultivars were inoculated with or without NSM and then grew for 14 days in gnotobiotic bottles. (A and B) Properties of the soil used in this study. (A) Photos of the four soil samples that were used to test rice-microbiota interactions. (B) The radar plot showing the physical and chemical properties of 4 soils. A total of 12 parameters of soil physical and chemical properties were measured and the relative values (%) of each parameter relative to black paddy soil are plotted. (C−E) Effects of NSM on the root growth of 10 cultivars. (C) Subspecies on 10 different rice cultivars. (D) Comparison of the lengths of the roots of 10 rice cultivars inoculated with (+) or without (-) NSM. The data were collected from 4–5 biological replicates. Error bars, standard deviations; ns, not significantly different; *, **, and *** indicate *p* < 0.05, 0.01, and 0.001, respectively (Student’s *t* test, n = 8−23). -, inoculated with filter membrane-sterilized NSM; +, inoculated with live NSM. The information for the 10 cultivars is available from RiceData (<https://www.ricedata.cn/variety/>). The full names and abbreviations of the rice cultivars are as follows: MH63, Minghui63; ZS97, Zhenshan97; 9311, 93-11; KIT, Kitaake; HY, Hwayoung; NIP, Nipponbare; NG9108, Nangeng9108; NG46, Nangeng46; ZH17, Zhonghua17; and ZH11, Zhonghua11.


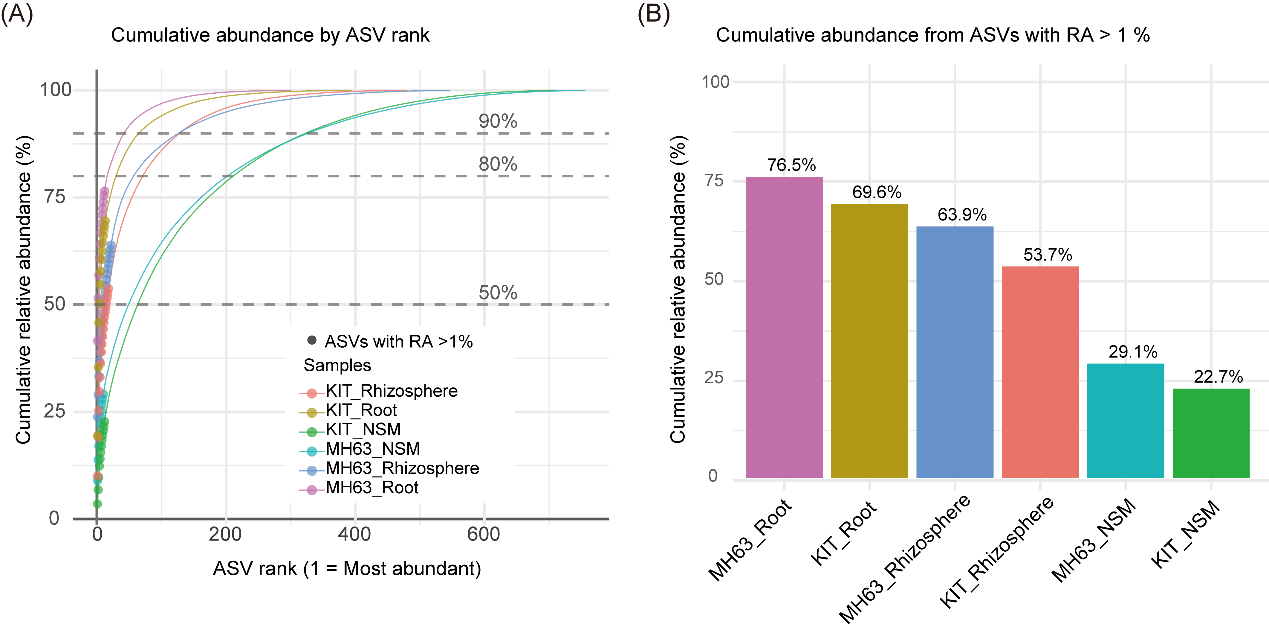


**Figure S3 Analysis of ASV rank-abundance relationships for core microbiota definition.** (A) Cumulative abundance curves showing the relationship between ASV rank (ordered from most to least abundant) and cumulative relative abundance. Curves represent individual samples, with points highlighting ASVs ≥ 1% relative abundance. (B) Quantitative assessment of the 1% cutoff, showing the percentage of total community abundance accounted for by ASVs with relative abundance ≥ 1%. These two plots were drawn using data from Table S1.


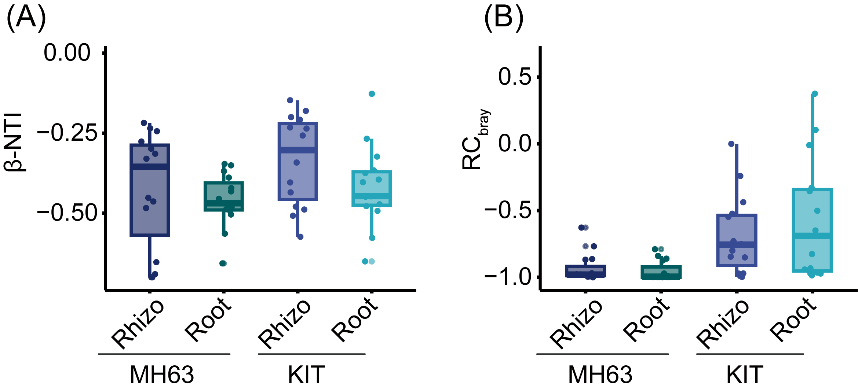


**Figure S4 Inferring the ecological processes of community assembly in gnotobiotic cultivation.** The ecological processes contributed to microbiota assemblage were inferred based on the β-mean-nearest-taxon-distance indexes (βNTI) (A) and Bray-Curtis-based Raup-Crick indexes (RC_bray_) (B). |βNTI| < 2 denotes stochastic processes governing the community assemblage. For stochastically assembled communities, RC_bray_ < -0.95 denotes homogenized dispersal dominating the community assemblage; |RC_bray_| < 0.95 denote ecological drift dominating the community assemblage. Each dot represents the β-NTI (A) or RC_bray_ calculated from each pairwise sample.


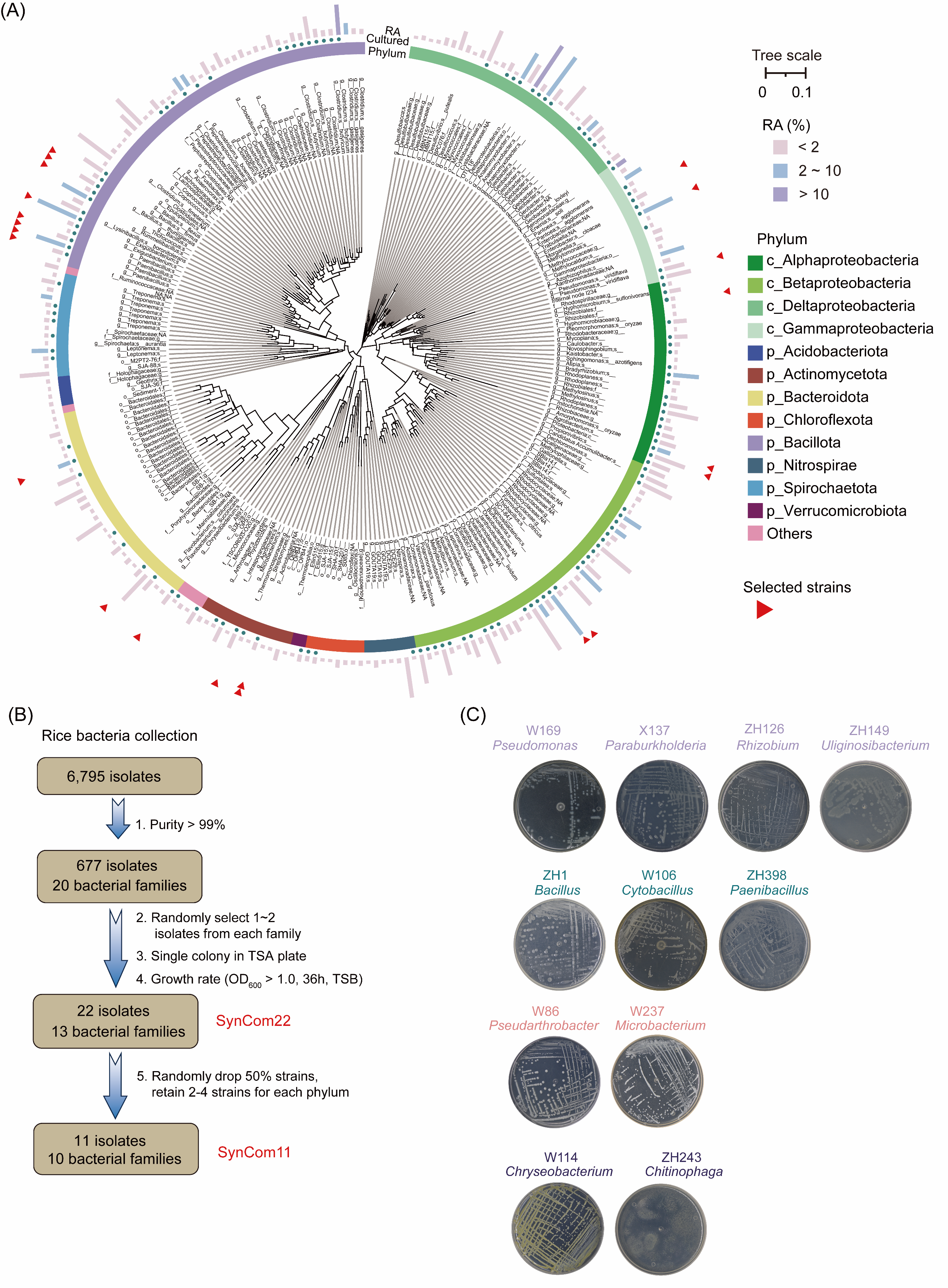


**Figure S5 Schematic diagram depicting the bottom-up design of SynCom22 and simplification from SynCom22 to SynCom11.** (A) Collection of bacterial isolates from the rice root microbiota. The inner ring represents the phylogenetic tree of the bacterial ASVs detected in MH63 rice roots. The 2^nd^ ring represents the bacterial taxonomy at the phylum level, whereas the Pseudomonadota are divided into classes. The 3^rd^ ring indicates the bacterial isolates collected in this study (cultured). The outer ring represents the relative abundance (RA) of bacterial taxa detected in MH63 roots. (B) The procedure for SynCom11 design. In high-throughput cultivation using 96-well plates, the purity of a bacterial isolate was defined as the percentage of its most abundant 16S rRNA gene sequence variant within a single well. (C) Photos of colonies of the 11 bacterial strains in SynCom11.


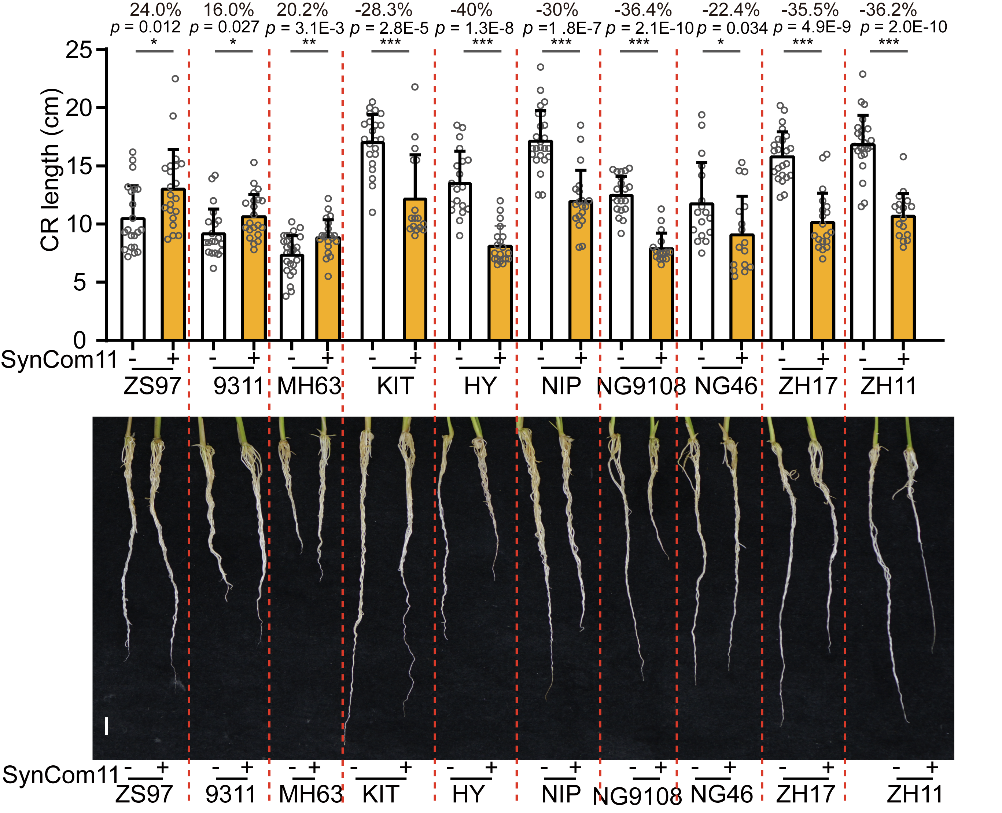


**Figure S6 Effects of SynCom11 on root growth of 10 cultivars.** Comparisons of the crown root (CR) lengths of 10 cultivars after SynCom11 inoculation. The relative root growth (%) and *p* values are shown in the plots (Student’s *t* test, n = 14−27). Bar = 2 cm. Error bars, standard deviation; *, **, and *** indicate *p* < 0.05, 0.01, and 0.001, respectively. -, heat-killed SynCom; +, live SynCom.


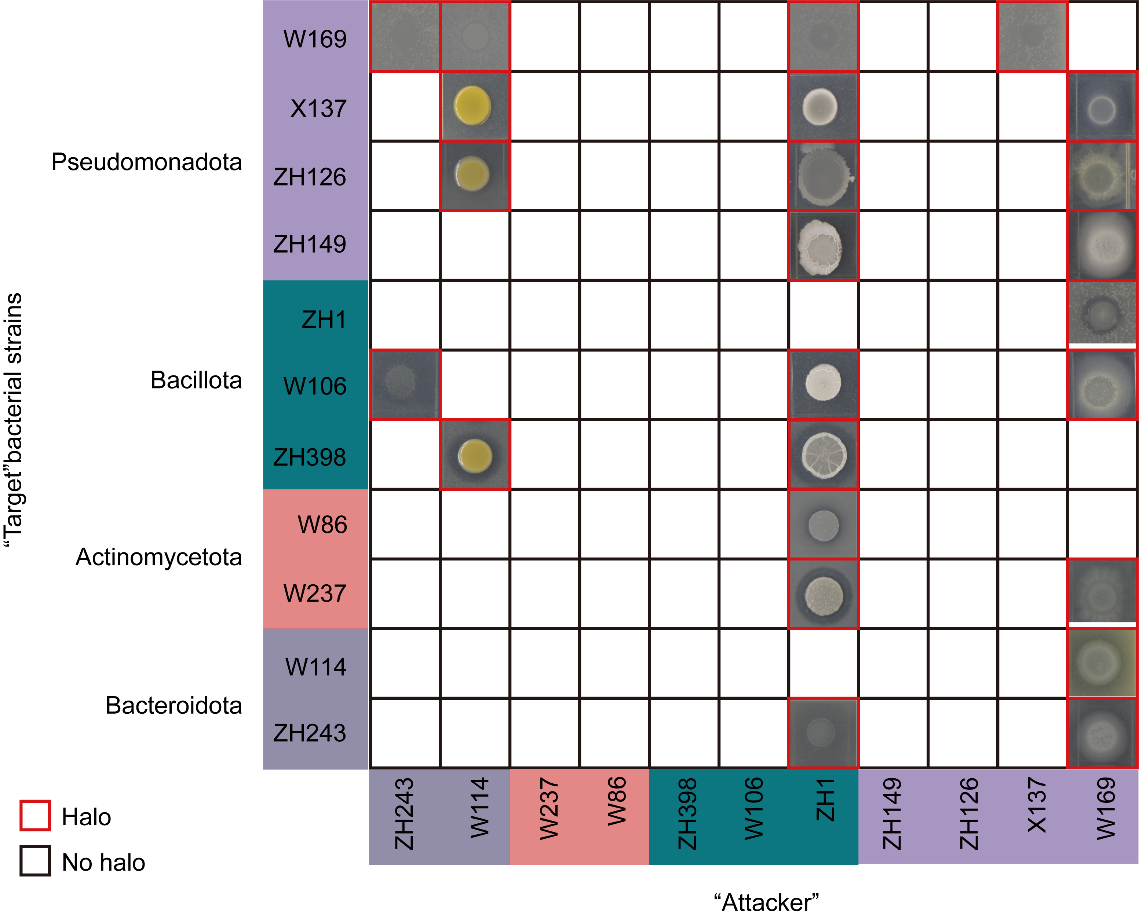


**Figure S7 Binary inhibition assays between 11 strains on 0.1 × TSA plates.** The target bacterial strains are presented along the horizontal axis, whereas the attacker bacterial strains are listed vertically. The red boxes indicate clear halos, and photos of the bacterial colonies are shown. The experiments were repeated twice, and the same results were obtained.


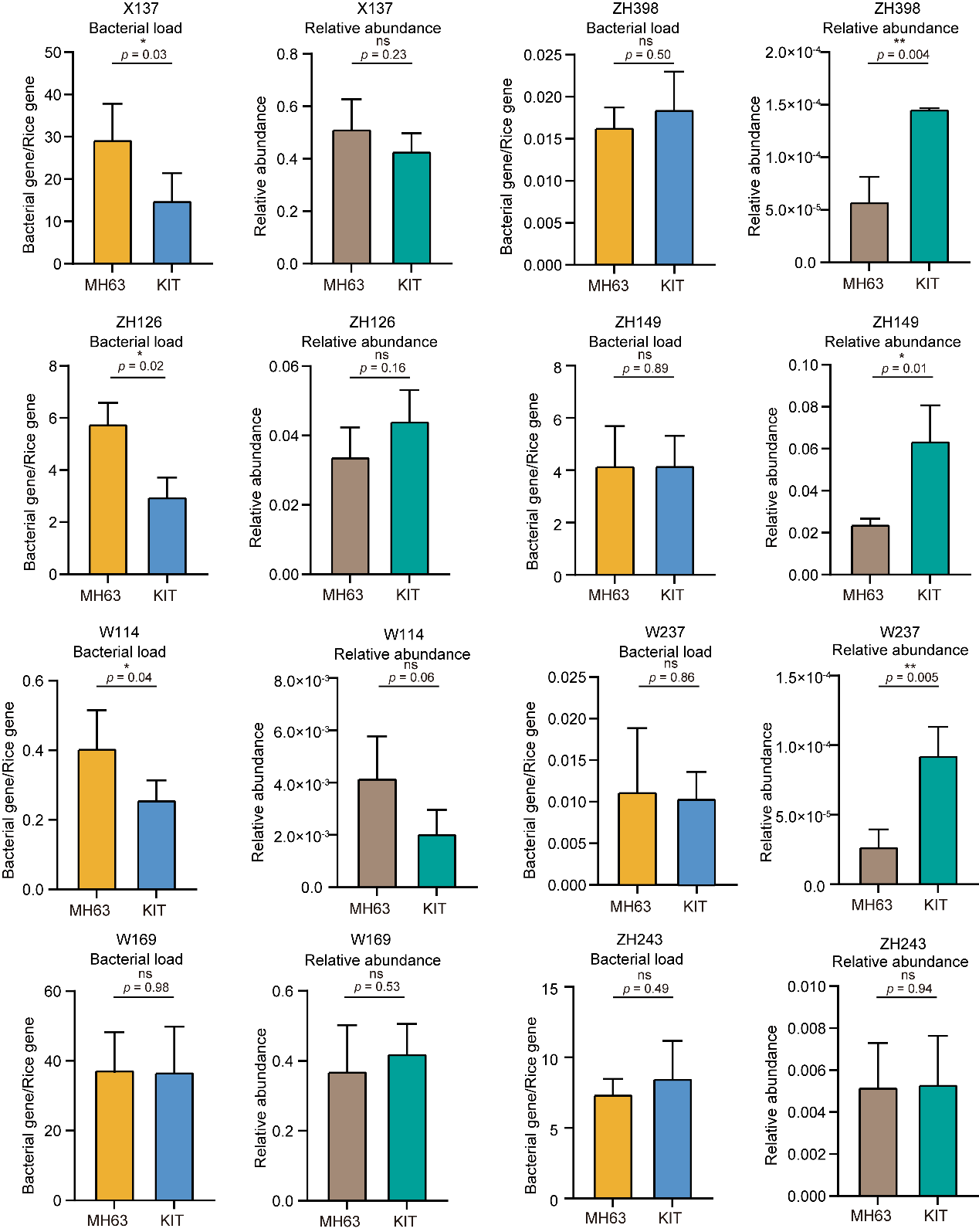


**Figure S8 Absolute and relative quantification of bacterial load in rice roots.** MH63 and KIT inoculated with SynCom11 and root samples were collected at 14 days post-SynCom inoculation. Bar plots showing the absolute and relative abundance of strains in the roots of MH63 and KIT plants. The bacterial loads (bacterial gene/rice gene) were calculated by qPCR-based absolute quantification of bacterial genes and rice genes. The relative abundance (RA) of each strain was analyzed via 16S rRNA gene amplicon sequencing. Data are presented as mean ± SD (n = 3−5 biological replicates). ns, no significant difference; * and **, *p* < 0.05 and 0.01, respectively (Student’s *t* test).


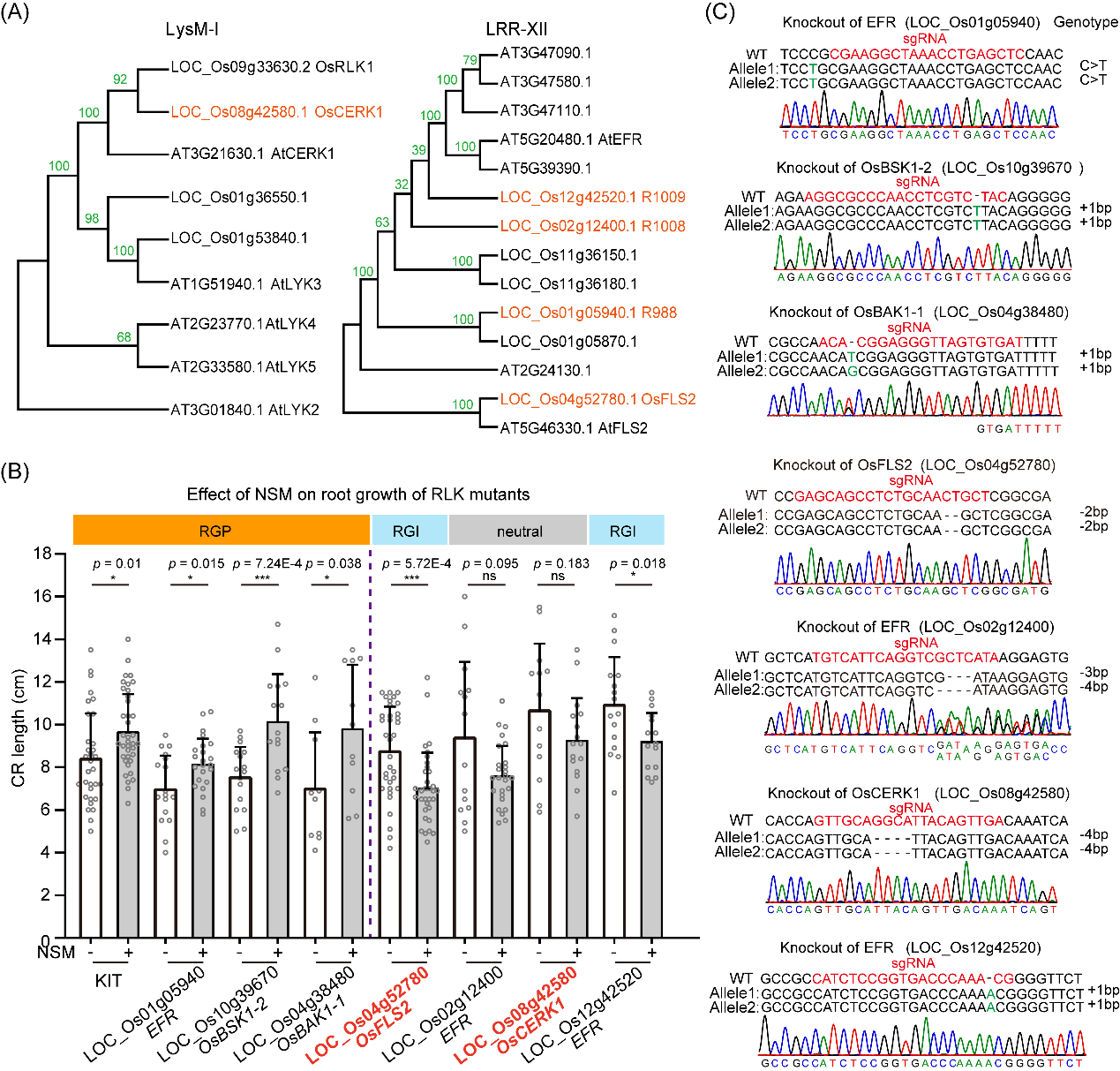


**Figure S9 Screening of 2 subgroups of RLKs involved in NSM-mediated regulation of root growth.** (A) Phylogenetic analysis of subgroup I of LysM RLKs and subgroup XII of LRR-RLKs. The full-length amino acid sequences were aligned via ClustalW, and a neighbor-joining tree was constructed via MEGA7.0 with 1000 bootstrap replicates. The numbers at the branches indicate the bootstrap values. R988, R1008, and R1009 represent the IDs of three EFR-like RLKs in the CRISPR/Cas9 genome editing library. (B) Effects of NSM on RLK mutants and wild-type KIT. Bar plots showing the comparison of the crown root (CR) lengths of 7 RLK gene mutants and WT plants inoculated with or without NSM for 14 days. The data are from 2 experiments with 4 to 6 biological replicates each. Error bars, standard deviation. ns, no significant difference; * and *** indicate *p* < 0.05 and 0.001, respectively (Student’s *t* test, n = 9−39). (C) Genotyping of 7 RLK mutants via Sanger sequencing. The gRNA guide sequences are shown in red.


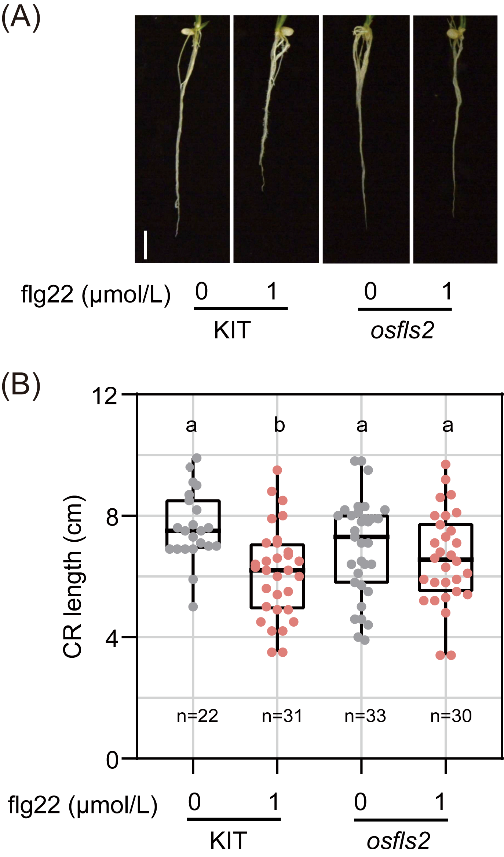


**Figure S10 *Osfls2* mutant impairs flg22-triggered root growth inhibition.** Germ-free germinated rice seeds were grown in 0.5 × MS media supplemental with 0 and 1 μM of flg22 peptide. The root length was examined at 7 days post inoculation. Different letters indicate statistically significant differences (one-way ANOVA followed by Duncan’s multiple range test, *p* < 0.05). Bar = 1cm (A).


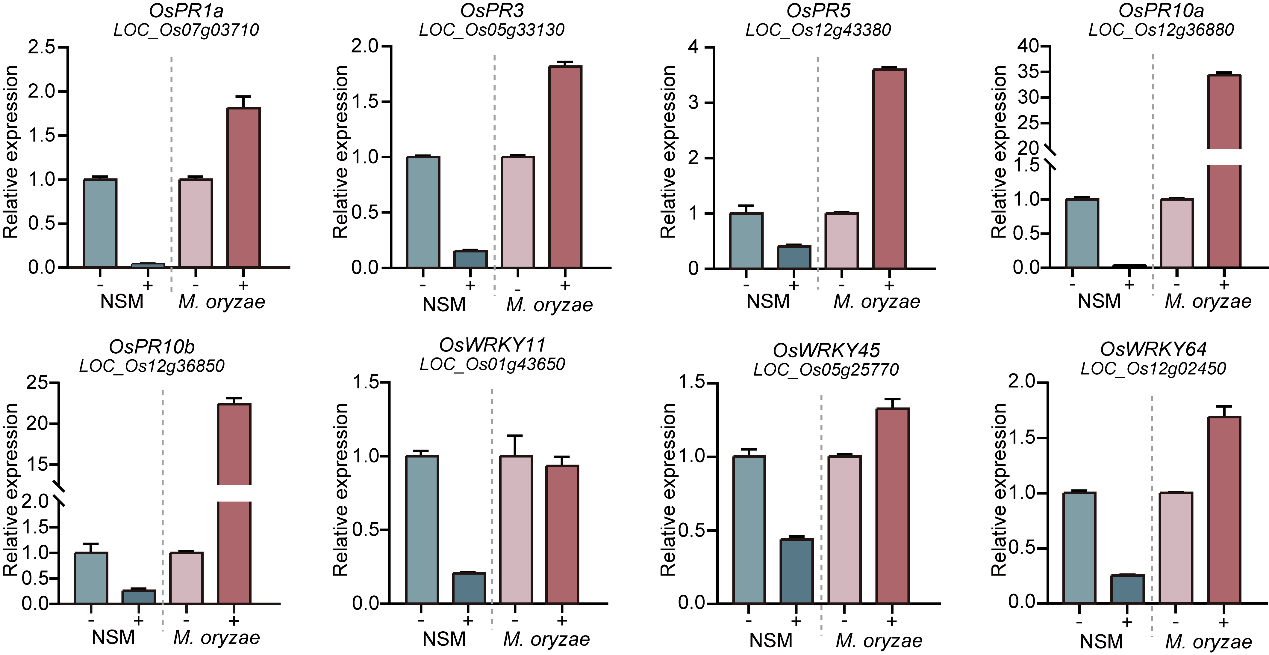


**Figure S11 Suppression of rice defense-related genes by NSM inoculation.** Comparisons of *PR* and *WRKY* gene expression in rice roots after NSM and *M. oryzae* inoculation of germ-free rice (cv. KIT) in gnotobiotic bottles. The relative gene expression levels were determined via RT-qPCR using *OsUBQ10* as a reference gene. The error bars represent the standard deviation (n = 3 technical replicates).


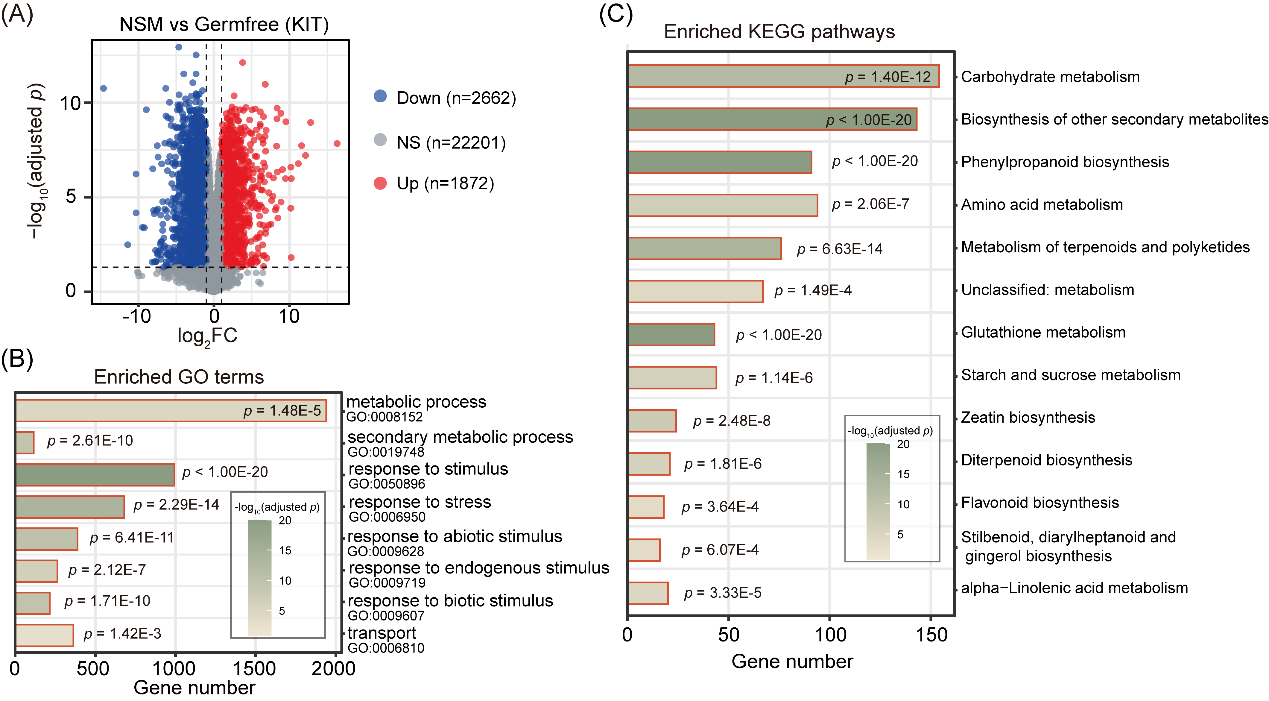


**Figure S12 Rice genes responding to NSM-inoculation in KIT roots.** (A) Volcano plot showing downregulated and upregulated DEGs after NSM inoculation in KIT roots (fold change ≥ 2, adjusted *p* value (*p*-adj) < 0.05). NS, no significant change. (B) Enriched GO terms and KEGG pathways of KIT DEGs. Benjamini-Hochberg adjusted *p* value.


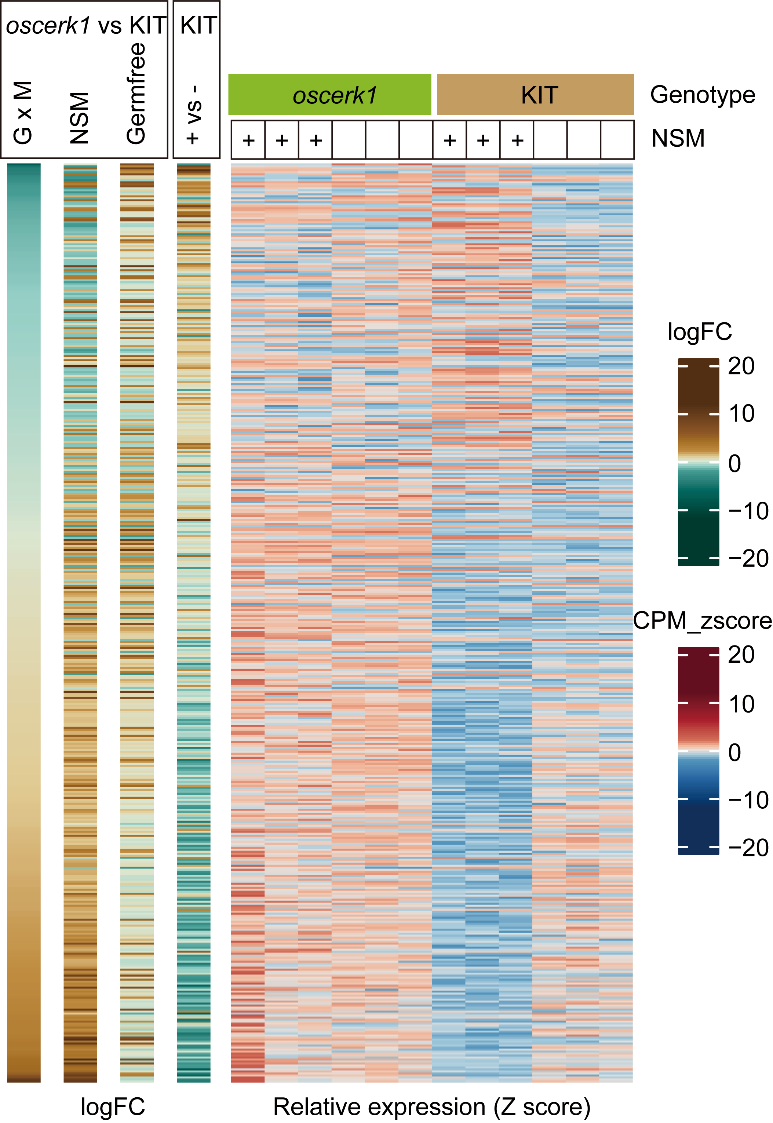


**Figure S13 Heatmap showing the fold changes and normalized expression of *oscerk1*-associated DEGs.** logFC, log_2_-fold change; + vs -, fold changes of genes after NSM inoculation in wild type (KIT); z score, median-centered Z score of expression value.
